# Overlap in oncogenic and pro-inflammatory pathways associated with areca nut and nicotine exposure

**DOI:** 10.1016/j.cpt.2023.09.003

**Published:** 2023-09-17

**Authors:** Krati Garg, Anuj Kumar, Vidisha Kizhakkethil, Pramod Kumar, Shalini Singh

**Affiliations:** a Department of Biochemical Engineering and Biotechnology, Indian Institute of Technology (IIT) Delhi, Delhi 110016, India; b Division of Molecular Biology, ICMR-National Institute of Cancer Prevention and Research (ICMR-NICPR), Indian Council of Medical Research, Noida, Uttar Pradesh 201301, India; c Department of Biotechnology, Vellore Institute of Technology (VIT), Vellore, Tamil Nadu 632024, India; d ICMR-National Institute of Cancer Prevention & Research (ICMR-NICPR), Indian Council of Medical Research, Noida, Uttar Pradesh 201301, India

**Keywords:** Cancer, Areca nut, Nicotine, Interactome, Cell signaling

## Abstract

**Background:**

Betel nut/areca nut/*Areca catechu* is one of the most commonly used psychoactive substance, and is also a major preventable cause of cancer. Unlike other psychoactive substances, such as nicotine, the mechanisms underlying addiction to areca nuts and related oncogenesis remain elusive. Recent reports suggest a possible overlap in the mechanisms of action of nicotine and areca nuts in the human body. Thus, this study aimed to investigate the interactome of human proteins associated with areca nut exposure and the intricate similarities and differences in the effects of the two psychoactive substances on humans.

**Methods:**

A list of proteins associated with areca nut use was obtained from the available literature using terms from Medical Subject Headings (MeSH). Protein-protein interaction (PPI) networks and functional enrichment were analyzed. The results obtained for both psychoactive substances were compared.

**Results:**

Given the limited number of common proteins (36/226, 16%) in the two sets, a substantial overlap (612/1176 nodes, 52%) was observed in the PPI networks, as well as in Gene Ontology. Areca nuts mainly affect signaling pathways through three hub proteins (alpha serine/threonine-protein kinase, tumor protein 53, and interleukin-6), which are common to both psychoactive substances, as well as two unique hub proteins (epidermal growth factor receptor and master regulator of cell cycle entry and proliferative metabolism). Areca nut-related proteins are associated with unique pathways, such as extracellular matrix organization, lipid storage, and metabolism, which are not found in nicotine-associated proteins.

**Conclusions:**

Areca nuts affect regulatory mechanisms, leading to systemic toxicity and oncogenesis. Areca nuts also affect unique pathways that can be studied as potential markers of exposure, as well as targets for anticancer therapeutic agents.

## Highlights

• The mechanisms of action of psychoactive agents, such as areca nuts and nicotine, overlap significantly.
• The molecular pathology of areca nuts involves the alteration of multiple signaling pathways.
• Signaling pathways are mainly dysregulated through three hub proteins (alpha serine/threonine-protein kinas, tumor protein 53, and interleukin-6), which are common between the areca nut and nicotine protein-protein interaction (PPI) networks, as well as two unique hub proteins (epidermal growth factor receptor and master regulator of cell cycle entry and proliferative metabolism).
• Extracellular matrix organization, lipid storage, and metabolism are unique pathways affected only by areca nut, but not by nicotine.

## Graphical abstract

**Figure:**
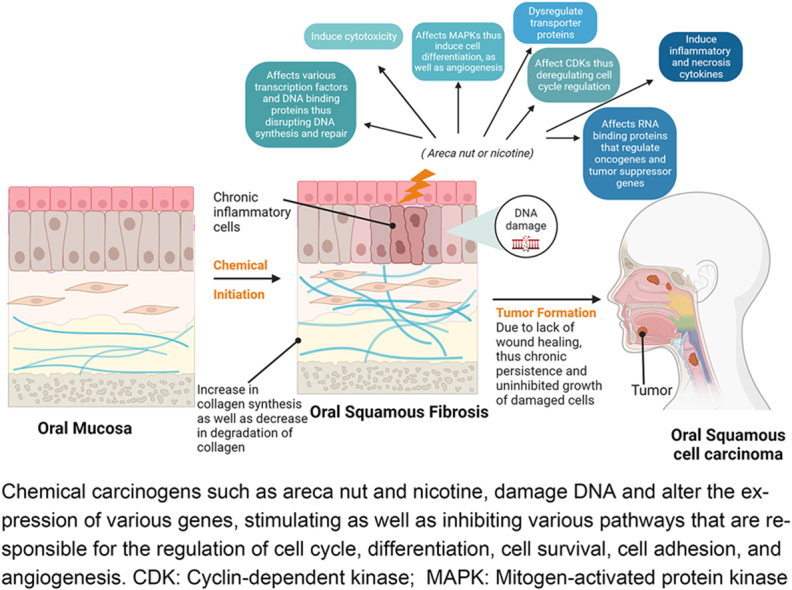

## Introduction

Tobacco and betel nut/areca nut/*Areca catechu* are the leading causes of cancer-related mortality and morbidity worldwide.^1^^,^^2^ Cancers caused by tobacco or areca nuts can be easily prevented by discontinuing their use. Although tobacco and areca nuts are classified as class I carcinogens by the International Agency for Research on Cancer (IARC), knowledge of the systemic effects of areca nuts is growing consistently.^3^, ^4^, ^5^

The use of areca nut has been widely promoted in sociocultural traditions. They are chewed as “betel quid” with tobacco as smokeless tobacco (SLT) or without tobacco as “pan masala” and mouth freshener.^6^^,^^7^ Areca nuts contain five alkaloids: arecoline, guvacine, guvacoline, arecaidine, and chavibetol. The principal alkaloid, arecoline, is metabolized to arecaidine by salivary enzymes, which is further metabolized into nitroso-derivatives.^8^ Nitroso-derivatives cause oral lesions and later oral submucosal fibrosis (OSF), which further leads to the development of oral squamous cell carcinoma (OSCC). Although multiple cellular pathways associated with areca nut addiction have been identified, the exact mechanisms underlying areca nut addiction and its correlation with oncogenesis remain elusive.^6^^,^^9^^,^^10^ Currently, there are no reliable biomarkers of areca nut exposure. Unlike the alkaloid markers of tobacco exposure, areca nut alkaloids have a short half-life of less than half a day.^11^^,^^12^ Thus, there is a pressing need to identify host markers of areca nut exposure, especially differential markers between areca nut and tobacco use.

Arecoline is a known muscarinic receptor agonist; however, its nicotinic activity has recently been reported, which might be a common reason for addiction and other similar effects caused by both arecoline and nicotine.^6^^,^^13^^,^^14^ Similarly, nicotine can cause non-areca nut OSF.^15^ Thus, there is a possible overlap between the mechanisms of action of areca nuts and tobacco in the human body, which needs to be investigated in detail.

To understand the role of areca nuts in carcinogenesis and other ailments due to toxicity, a list of human proteins associated with areca nuts was prepared from the available literature. The functions of proteins associated with areca nuts were comprehensively analyzed using a system-based approach and more than one functional enrichment tool for a random non-weighted set of proteins.^16^ The present data on proteins associated with areca nuts and their alkaloids do not provide a specific association with any alkaloid; therefore, proteins are considered to be associated with areca nuts only. The data were compared with data on proteins associated with nicotine, which is the principal alkaloid in tobacco. By comparing the data, the overlapping mechanisms of two alkaloids in oncogenesis and pro-inflammatory roles were analyzed. Unique pathways associated with areca nut were also identified, which could be crucial in the identification of potential biomarkers.

## Methods

### Database preparation

A list of genes associated with areca nut exposure was prepared using Medical Subject Headings (MeSH) terms in the PubMed database. The MeSH term “areca nut,” its synonym “betel nut,” and the scientific name “*Areca catechu*” were used along with the term “gene.” The following MeSH terms were formed: (areca nut OR betel nut OR *Areca catechu*) AND (genetics), (areca nut OR betel nut OR *Areca catechu*) AND (gene), and (areca OR betel) AND (gene). MeSH terms were optimized based on the number of relevant hits i.e., the literature available in a single year (2019–2020). The terms (areca nut OR betel nut OR *Areca catechu*) AND (gene) were optimal. The optimized MeSH term was then used in the PubMed database, and 415 pieces of literature were retrieved up to 07/01/2022. We manually identified 226 genes associated with areca nuts from the available literature. Genes related to organisms other than *Homo sapiens* were excluded from the list. The UniProt and Entrez IDs of these genes were retrieved using the UniProt retrieval tool, and a database was formed.^17^

The list of nicotine-associated proteins was prepared using the STITCH database by entering the terms “nicotine” and “*Homo sapiens*” organism.^18^ The interaction sources and confidence level (0.400) were set at default, whereas the stereoisomers were shown as separate compounds. The maximum number of nicotine interactors was set to a custom value of 200.

### Construction and analysis of protein-protein interaction networks

The protein-protein interaction (PPI) networks for areca nut and nicotine were constructed using the STRING database in Cytoscape software (v.3.8.2, https://cytoscape.org/download.html) with the help of the StringApp plugin and a default confidence score cut-off of 0.40 (medium confidence).^19^ The STRING database is the source of the STITCH database. Nicotine-associated proteins were mapped onto the PPI network of areca nut-associated proteins.

The topological parameters of the PPI networks were analyzed using the Cytoscape software network analyzer tool. The networks were analyzed as undirected networks. The computed topological parameters included the number of neighbors and edges, average number of neighbors, diameter, and radius of the networks, characteristic path length, clustering coefficient, network density, heterogeneity and centralization, and connected components. The computed node parameters included the average shortest path lengths, clustering coefficients, centrality of closeness, eccentricity, stress, node degrees, centrality of neighbors, neighborhood connectivity, topological coefficient, and radiality.

Betweenness centrality *vs*. node degree graphs for the networks were obtained via topological analysis using the CyChart plugin. The CytoHubba plugin was used to confirm and visualize hub genes based on the node degree and centrality of the relationship.

The Molecular Complex Detection (MCODE) plugin was used to identify protein clusters (protein complexes) present in the network. The set of proteins associated with areca nuts and the proteins in the clusters were then phenotypically analyzed using the web-based Gene ORGANizer tool, which links genes to organs, body systems, body regions, and germ layers.^20^

### Functional enrichment analysis

Functional enrichment was performed on the areca nut (*n* = 226) and nicotine (*n* = 200) proteins. Protein sequences were obtained using the UniProt retrieval system and entered into subcellular localization prediction with the annotation of functional Gene Ontology annotation (CELLO2GO) to determine the distribution of proteins in subcellular compartments.^21^

Protein Gene Ontology (GO) was determined using the Protein Analysis Through Evolutionary Relationships (PANTHER; http://www.pantherdb.org/), Database for Annotation, Visualization, and Integrated Discovery (DAVID v6.8; https://david.ncifcrf.gov/home.jsp), and FunRich (http://www.funrich.org/) databases.^22^, ^23^, ^24^ Gene names were converted to UniProt IDs using the UniProt Retrieve/ID mapping tool. DAVID was used to predict gene function based on Kyoto Encyclopedia of Genes and Genomes (KEGG) pathways, Online Mendelian Inheritance in Man (OMIM) disease enrichment analysis, Simple Modular Architecture Research Tool (SMART), and InterPro domain analysis.^23^

Comparative functional profiling was performed using the g: GOST tool in g: Profiler (https://biit.cs.ut.ee/gprofiler/gost).^25^ The g: GOST tool was used for functional enrichment analysis and comparison of the set of proteins associated with areca nut and nicotine-associated proteins at *P* values < 10^−9^ for *Homo sapiens*.

Relationships between proteins based on their signal transduction pathways were determined using the SIGnaling Network Open Resource (SIGNOR) (https://signor.uniroma2.it/) database. The query proteins were analyzed by selecting the “Connect” option and *Homo sapiens* species; the first neighbors were not included.^26^

## Results

### Protein-protein interaction network analysis

**Figure 1:**
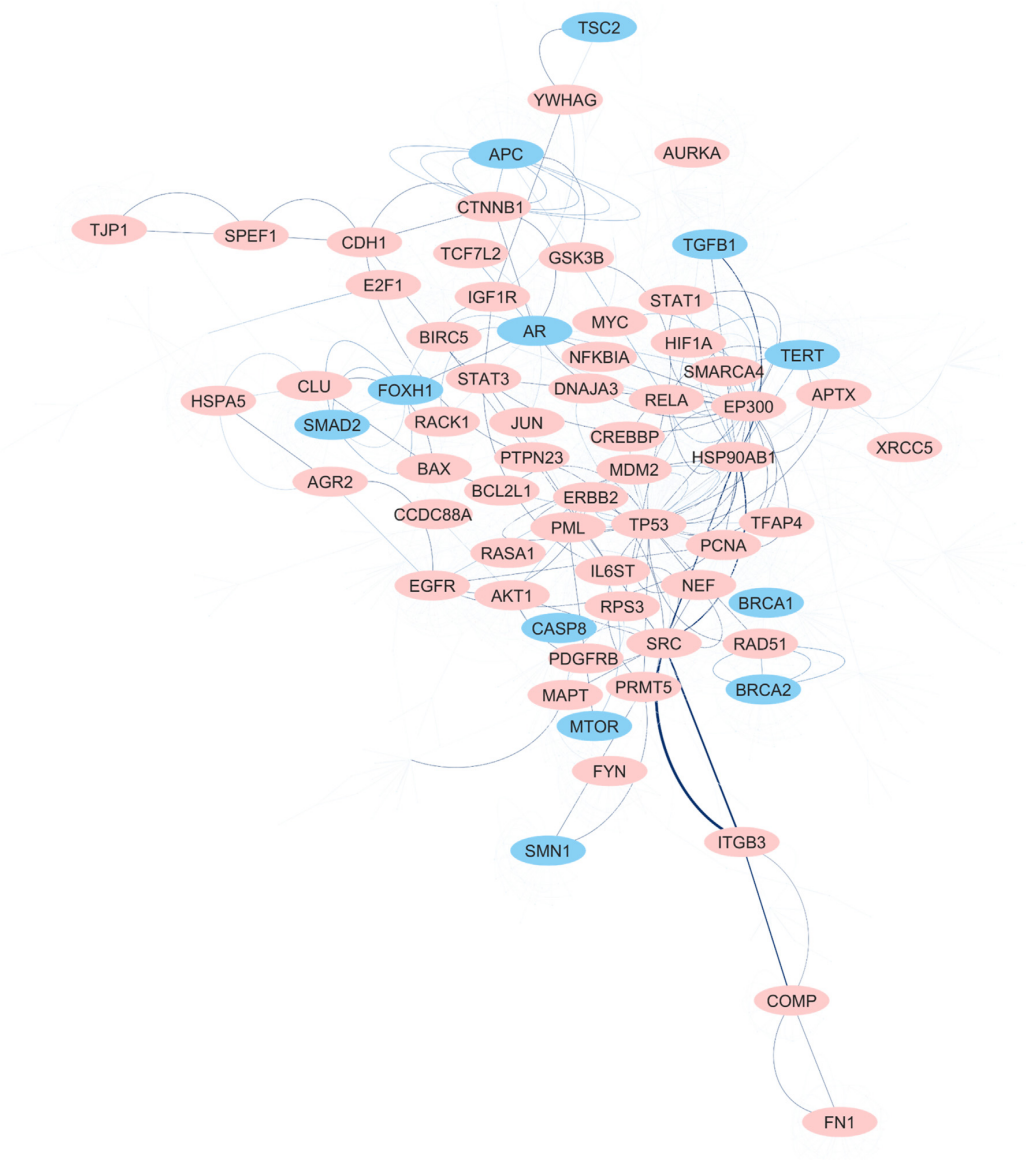
Hub proteins (nodes) common between the areca nut and nicotine networks. Common proteins (nodes) are colored in red, and hub proteins (nodes) unique in areca nut are colored in blue. The unique hub nodes include Adenomatous polyposis coli protein (APC), Tuberin (TSC2), Mothers against decapentaplegic homolog 2 (SMAD2), Forkhead box protein H1 (FOXH1), Androgen receptor (AR), Transforming growth factor beta-1 proprotein (TGFB1), Telomerase reverse transcriptase (TERT), Caspase-8 (CASP8), Serine/threonine-protein kinase (mTOR), Breast cancer type 1 susceptibility protein (BRCA1) and Breast cancer type 2 susceptibility protein (BRCA2).

A set of 226 proteins associated with areca nut was systematically identified from the literature using the MeSH term, and another set of 200 proteins associated with nicotine was obtained from the STITCH database. In comparison, only 16% (*n* = 36) of nicotine-associated proteins were common with the areca nut-associated proteins. Although the difference in topological parameters of the network was negligible, mapping nicotine-associated proteins into the PPI network of areca nut-associated proteins yielded a 52% overlap (*n* = 612/1176 nodes) [Figure 1; Supplementary Table 1]. However, on the deletion of overlapping nodes, the 52 connected components of the network broke down into 218 connected components, suggesting that overlapping nodes form the core of the network in the absence of those networks falling apart.

The degree *vs.* betweenness centrality graph of areca nut-associated proteins showed that nodes with a high-degree centrality (>100) had a significant betweenness centrality, whereas nodes with a low-degree centrality (<70) had a near-zero betweenness centrality value [Supplementary Figure 1].^27^ The graph also suggested five nodes that had a high node degree and betweenness centrality, eliminating nicotine nodes from the nicotine PPI network. These proteins also showed the highest closeness centralities. They were identified as the five main hub proteins, namely epidermal growth factor receptor (EGFR), master regulator of cell cycle entry and proliferative metabolism (MYC), alpha serine/threonine-protein kinase (AKT1), tumor protein 53 (TP53), and interleukin-6 (IL-6), in the areca nut PPI network and TP53, AKT1, IL-6 and FOS/JUN (activator proteins) in the nicotine PPI network [Supplementary Figure 1].

Next, 48.3% (226) of the proteins associated with areca nut and 30.6% (200) of the proteins associated with nicotine were predicted to localize in the cell nucleus [Supplementary Figure 2]. Other proteins associated with areca nut were present mainly in the cytoplasm (21.1%) and extracellular components (20.3%), whereas proteins associated with nicotine were present in the extracellular components (19.2%) and cytoplasm (17.8%). Compared with the 26.4% of nicotine-associated proteins localized to the plasma membrane, the percentage of areca nut-associated proteins was very low.

### Cluster analysis

The MCODE plugin suggested 8 clusters in the areca nut PPI network, with cluster 1 being the largest [Supplementary Table 2] with 63 nodes. The smallest clusters, 7 and 8, had four nodes each. A total of 131/226 genes were cumulatively present in these clusters [Supplementary Table 2]. Six clusters were obtained for the nicotine PPI network, with clusters 2 (29 nodes), 1 (27 nodes), and 6 (three nodes) being the largest. Collectively, 92/200 genes were present in all these clusters [Supplementary Table 3].

**Figure 2:**
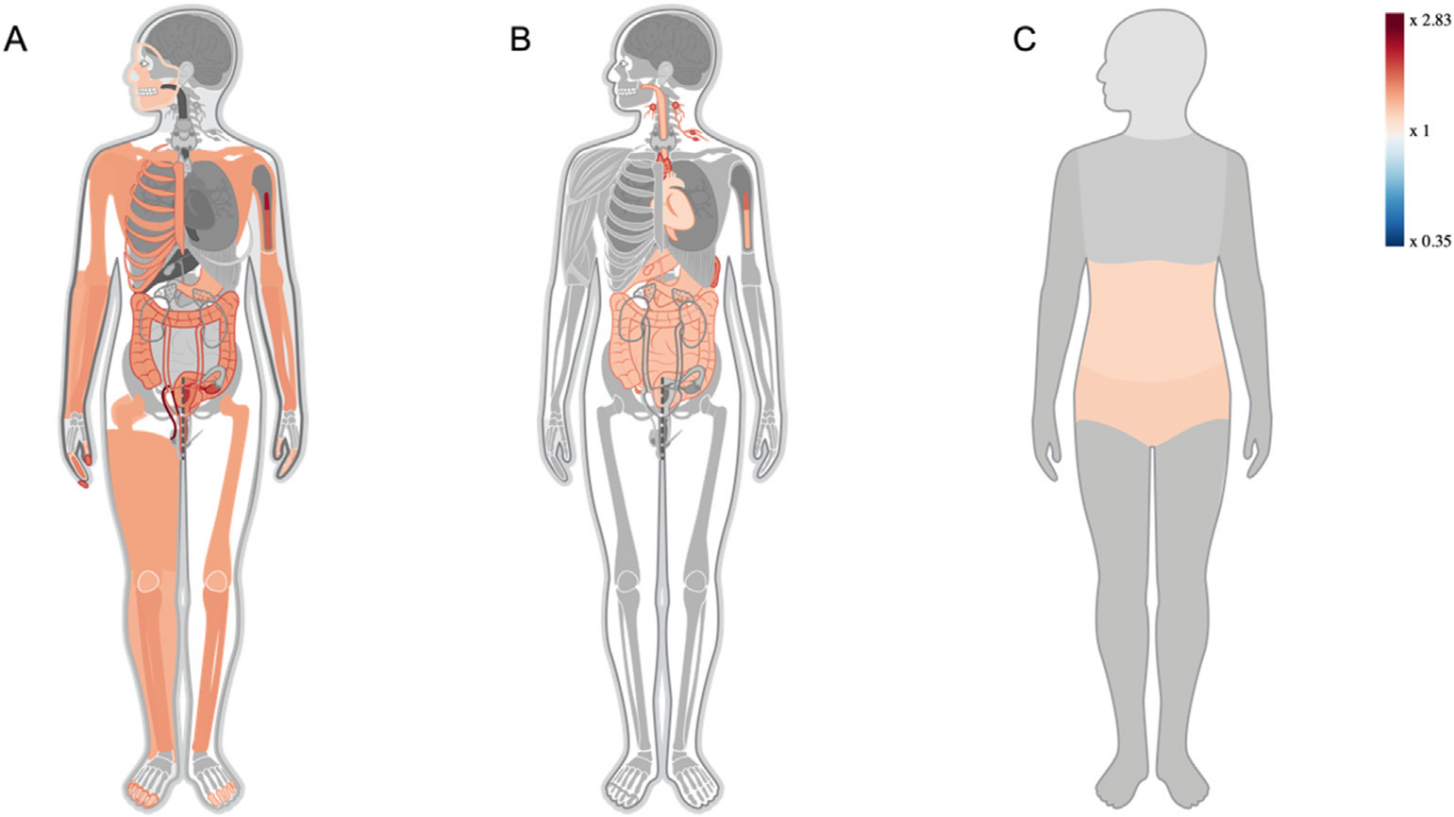
Phenotypic analysis of areca nut PPI network. Protein enrichment in (A) organs, (B) regions, and (C) systems. The heat map shows the level of enrichment i.e., darker red color indicates higher enrichment. PPI: Protein-protein interaction.

The Gene ORGANizer data of proteins associated with areca nuts showed protein enrichment in 44 organs (x > 1.24, x stands for fold increase in comparison to the normal level of the protein) and a significant decline in one organ, the cranial nerves (x0.5, *P* = 0.000138) [Figure 2]. Similarly, protein enrichment was observed in four systems – digestive (1.2966, *P* = 0.000502), immune (x1.3838, *P* = 0.000462), lymphatic (x1.6135, *P* = 0.001900), and cardiovascular (x1.1895, *P* = 0.011100) systems. The pelvis (x1.2403, *P* = 0.004940) and abdomen (x1.1976, *P* = 0.009040) showed maximum enrichment [Figure 2]. Among the areca nut-associated proteins, phenotypic analysis of all clusters showed that cluster 1 affected most body parts, with 42 of 63 proteins in cluster 1 showing enrichment. Among nicotine-associated proteins, 17 of the 27 proteins in cluster 1 were enriched [Supplementary Table 4]. The most enriched areca nut-associated proteins in cluster 1 in these body parts were KRAS, CDKN1B, CDKN1A, AKT1, TGFB1, STAT1, and STAT3 [Supplementary Table 5]. The most enriched nicotine-associated proteins in cluster 1 in these body parts included AKT1, POMC, NFKB1, RAF1, TP53, and EIF2AK3 [Supplementary Table 6].

### Functional enrichment analysis

A comprehensive functional enrichment analysis was performed. According to PANTHER-based GO analysis, 226 proteins associated with areca nuts were involved in oxidative stress responses, nicotinic acetylcholine receptor signaling pathway, vasopressin synthesis, pantothenate biosynthesis, leucine biosynthesis, methylmalonyl pathway, alanine biosynthesis, and 5-hydroxytryptamine biosynthesis. GO analysis of the 200 nicotine-associated proteins yielded similar results.

In total, 223 of the 226 areca nut-associated proteins were classified using the PANTHER system. The proteins belonged to 19 protein families, mainly transcriptional regulators and protein-modifying enzymes; seven molecular functions mostly related to binding, catalytic activity, and molecular function regulator; 16 biological processes mostly involved in biological regulation, cellular process, and metabolic process; 54 pathways; and three cellular components, mostly cellular anatomical entities [Supplementary Figure 3]. In comparison, 198/200 nicotine-associated proteins were classified into 18 protein families: seven molecular functions, including binding, catalytic activity, and molecular function regulator; 18 biological processes; 76 pathways; and three cellular components, most of which were cellular anatomical entities (65.7%) [Supplementary Figure 4]. The function of nicotine-associated proteins was the same as that of areca nut-associated proteins.

OMIM disease classification of the areca nut-associated proteins suggested that 18 proteins were associated with 16 OMIM diseases [Supplementary Figure 5]. In addition, KEGG pathway enrichment was assessed, where 158 genes were annotated using 116 terms. They were then grouped into nine clusters, with cluster 1 obtaining a maximum enrichment score of 13.36. Sixty-six KEGG terms were not included in any of the nine clusters [Supplementary Figure 5]. In comparison, OMIM disease classification of nicotine-associated genes attributed 6/200 genes to three OMIM disease terms. KEGG pathway enrichment annotated 147 genes to 115 KEGG terms. These terms were then grouped into 12 clusters, with cluster 1 obtaining a maximum enrichment score of 7.01. A total of 67 KEGG terms were not included in any of the 12 clusters [Supplementary Figure 5].

The g: GOSt tool provided a comparative graphical representation of the functional enrichment of areca nut- and nicotine-associated proteins by detecting significantly enriched biological processes, pathways, regulatory motifs, and protein complexes. This included GO terms, KEGG pathways, Reactome and WikiPathways, regulatory motif matches from TRANSFAC, miRNA targets from miRTarBase, tissue specificity based on expression data from the Human Protein Atlas, protein complex data from the Comprehensive Resource of Mammalian Protein Complexes (CORUM) database, and human disease phenotype associations from Human Phenotype Ontology (HPO) [Supplementary Figure 6].

Enrichment of areca nuts and nicotine was also analyzed using the FunRich tool [Supplementary Figure 7]. Areca nut-associated proteins showed two biological processes – lipid storage and deoxyribonucleic acid (DNA) repair. In addition, a significant association was observed with transcription factor activity. Site of expression analysis of areca nut-associated proteins suggested their expression in the oral cavity, but not in the saliva [Supplementary Figure 8].

### Signaling interaction

Among the areca nut-associated proteins, 27/186 genes mapped in SIGNOR showed nuclear signaling, two of which coded major hub proteins - MYC and TP53. Another hub protein, EGFR, acts as a transmembrane receptor, whereas AKT1 signaling is cytoplasmic, and that of IL-6 is extracellular [Supplementary Figure 9A]. In comparison, 24/141 genes associated with nicotine mapped in SIGNOR were involved in nuclear signaling and encoded main hub proteins including JUN, FOS, and TP53 [Supplementary Figure 9B].

## Discussion

Areca nut-associated proteins were comprehensively analyzed using multiple enrichment tools to avoid database and methodology bias.^16^ The role of areca nuts in OSF and OSCC has been well established.^28^ In addition, OMIM disease terms associated with areca nut-related proteins are correlated with diseases, such as epidermolysis bullosa (EB) and fibrinogen-related blood disorders. EB simplex is a rare disease that increases mucosal fragility, resulting in blister formation, even after minor mechanical trauma. This disease can increase susceptibility to squamous cell carcinoma.^29^^,^^30^ Similarly, a prognostic role of fibrinogen has been proposed for OSCC.^31^

This study confirms the systemic effects of areca nuts, specifically on the digestive, immune, system lymphatic, and cardiovascular systems [Figure 2].^32^ Areca nut is known to increase the level of reactive oxygen species (ROS), create hypoxia-like conditions, promote autophagy, and inhibit tumor suppression in cells.^33^ Similarly, areca nuts activate multiple cell signaling pathways [Supplementary Figs. 3 and 9]. A signaling network skeleton for areca nuts was prepared by creating a PPI network of areca nut-associated proteins. This network overlapped with the nicotine network, including common hub proteins [Figure 1; Supplementary Table 1].^27^ The degree *vs.* betweenness centrality graphs of the two networks suggest that the PPI network of areca nut-associated proteins was less interconnected than the nicotine-associated network and more connected through hub proteins [Supplementary Figure 1]. This indicates that the effects of areca nut on the regulatory (signaling) cell pathways are greater than those induced by nicotine.

Commonality also emerged among hub proteins in the two networks [Supplementary Figure 1]. Reports suggest that EGFR, MYC, AKT1, and TP53 are major hub proteins in the OSCC protein network.^34^^,^^35^ The roles of these hub proteins in uncontrolled cell proliferation in OSCC are also known.^36^^,^^37^

**Figure 3:**
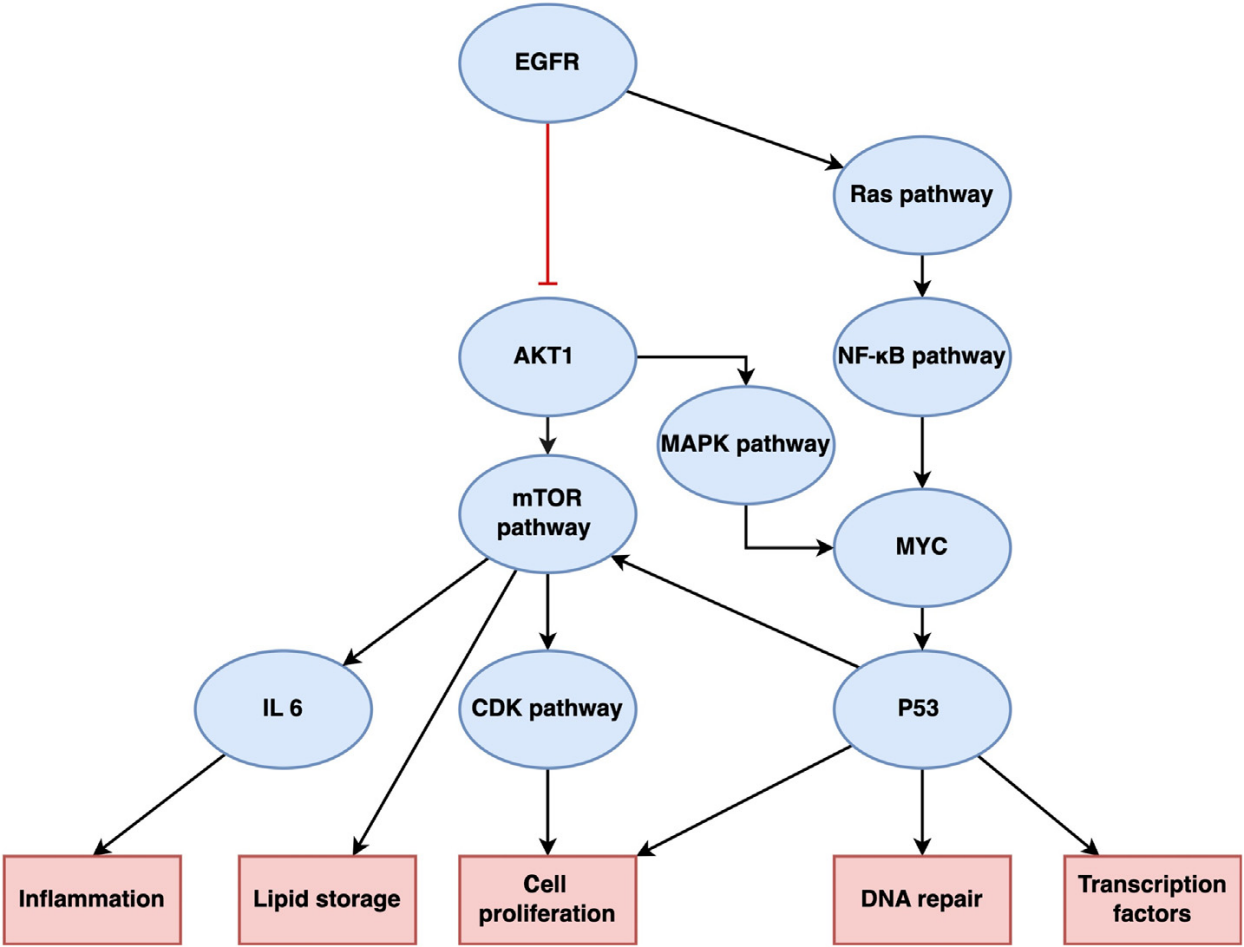
Schematic representation of core cell signaling pathways associated with areca nut use. Hub proteins and pathways are shown in blue elliptical boxes, and changes in cellular physiology are shown in red rectangular boxes. Signal directionality is shown using black arrows, and inhibition is shown as a red line. AKT1: Alpha serine/threonine-protein kinase; CDK: Cyclin-dependent kinase; EGFR: Epidermal growth factor receptor; IL6: Interleukin-6; MAPK: Mitogen-activated protein kinase; mTOR: Mammalian target of rapamycin; MYC: Master regulator of cell cycle entry and proliferative metabolism; NF-κB: Nuclear factor kappa light chain enhancer of activated B cells; Ras: Rat sarcoma.

The hub protein EGFR is activated by areca nuts, possibly via a stress-induced non-canonical kinase-dependent pathway [Figure 3].^38^ This further activates downstream signaling, such as the mitogen-activated protein kinase (MAPK) pathway directly, or indirectly via activation of the MYC protein (an oncoprotein).^39^ In addition, EGFR may suppress AKT1, which has antiproliferative activity and activates hub proteins. AKT1 suppression may affect multiple signaling pathways, such as activating one of two key catalytic pathways associated with areca nut proteins – “cyclin-dependent protein serine/threonine kinase (CDK) activity.” CDK activity was not observed in nicotine-associated proteins [Supplementary Figure 9]. CDKs have been proposed as potential biomarkers of OSCC.^40^ CDK4 and CDK6 overexpression in OSCC cells leads to cell cycle dysfunction. Similarly, areca nuts dysregulate CDK expression, mainly CDK1 and CDK2, thereby leading to alterations in cell cycle regulation.^41^ In our study, kinase regulation was only associated with areca nut-related proteins [Supplementary Figure 7]. AKT1 also activates mTOR, which is crucial for protein synthesis, glucose import, and lipid storage [Supplementary Figure 7]. Interruption of lipid storage and metabolism has been linked to carcinogenesis, as it leads to the dysregulation of cytokines and signaling pathways.^42^ The cholecystokinin receptor (CCKR) pathway, which plays a vital role in food intake and hunger regulation, was common and significant for both areca nuts and nicotine [Supplementary Figures 3 and 4]. CCKR has been associated with various human cancers, such as small-cell lung cancer, astrocytoma, pancreatic cancer, stromal ovarian cancer, and medullary thyroid carcinoma; however, its involvement in OSCC has not been reported yet.^43^ It also induces cyclin accumulation, CDK regulation, and downregulation of cell cycle inhibitors, which disrupts the cell cycle and benefits tumor cells.^44^ Another prominent pathway involves the gonadotropin-releasing hormone receptor, which regulates cellular proliferation. This pathway has been implicated in hormone-dependent tumors of the reproductive system, such as prostate, breast, and ovarian cancers, and in unrelated tumors, such as glioblastoma and lung, melanoma, and pancreatic cancers.^45^ Although this pathway has not been reported in OSCC pathogenesis yet, areca nut deregulates this pathway, suggesting its involvement in areca nut-induced carcinogenesis. Similarly, areca nuts may also activate other pathways through AKT1, including the rat sarcoma (Ras) and repressor/activator protein 1 (RAP1) signaling pathways, as determined via KEGG analysis [Supplementary Figure 5]. These pathways are common to both areca nuts and nicotine-associated proteins and are mainly involved in cell proliferation.

The hub protein MYC is possibly activated by MAPKs, which may be activated by stress induced by areca nuts.^46^ MAPK is a common areca nut- and nicotine-related protein associated with oral cancer progression and invasion.^47^ Areca nuts can activate MAPKs in OSCC, thereby aiding in tumor formation.^48^ MYC might also control the maximum number of proteins with transcriptional regulatory activity [Supplementary Figures 7 and 9]. Deregulation of various oncogenic transcription factors has been observed in OSCC, such as overexpression of NF-κB, c-Myc, STAT, Snail, β-catenin, and HIF1, as well as TP53 inactivation.^49^ The overexpression of other transcription factors, including E2F, SOX2, RUNX3, and Ets-1, is also observed in OSCC. These proteins are potential therapeutic targets for oral cancer.^50^ In addition to MYC, two hub proteins of the nicotine-associated PPI network, JUN, and FOS, were also present in the areca nut-associated PPI network [Supplementary Figure 1]. These proteins belong to the transcription factor activator protein (AP1) superfamily, and increased FOS expression and JUN nuclear expression have been observed during uncontrolled cell proliferation in OSCC tumors.^51^ AKT1 suppression also hampers the activity of the hub protein TP53, a master checkpoint inhibitor. Through TP53, areca nut impairs DNA mismatch repair and the nucleotide excision repair pathway in areca nut chewers with OSCC.^52^

Almost half of the proteins in the areca nut PPI network are localized in the nucleus (48%), where their activity is controlled by either TP53 or MYC, as most proteins exhibit “nucleic acid binding activity” and “transcription regulator activity” [Supplementary Figure 10]. Nuclear localization of areca nut-associated proteins was mainly involved in the upregulating transcription, whereas nuclear localization of nicotine-associated proteins was involved in suppressing transcription [Supplementary Figure 8]. Arecoline, the principal alkaloid of the areca nut, inhibits the DNA-binding activity of TP53, thereby suppressing the damage-specific DNA-binding protein (DDB2) and nucleotide excision repair pathways, contributing to areca nut-induced mutagenicity.^49^ This explains why DNA repair processes were the most prevalent biological process in enrichment analysis, and why they were not significantly related to nicotine-associated proteins [Supplementary Figure 7]. In addition, areca nuts induce cytotoxicity, cross-linking of DNA proteins, breakage of DNA strands, and unscheduled DNA synthesis (UDS) in oral keratinocytes and fibroblasts.^33^ In addition to DNA, areca nuts also affect the RNA-binding activity of proteins in an additional mechanism of areca nut-induced oral malignancy.^53^ Most areca nut-associated proteins localized in the nucleus were transcription factors, except for *CCND1-*encoded cyclin D1 (CD1). Thus, the most significant catalytic activity shown by areca nut-associated proteins was “transcription regulator activity” [Supplementary Figure 10]. Among the nuclear proteins, the roles of RUNX1, E2F1, MYOG, ETV6, SPI1, RELA, and NFATC1/2 in OSCC require further investigation. The nuclear proteins associated with nicotine were also mainly transcription factors, except for NR3C1 and PPARG (or NR1C3), which act as transcription factors and nuclear receptors, respectively. Nuclear genes, such as *FOS, JUN, MYC, TP53, EGR1, RUNX2, HIF1A, NFKB1*, and *SNAI1*, were involved in the signal transduction of both areca nut and nicotine, consolidating the overlap in nuclear signaling [Supplementary Figure 9]. Other oncoproteins in the nucleus, such as Ras, PCNA, and STAT3, also play roles in oncogenesis and cancer progression under the influence of extracellular signaling [Supplementary Figure 9].^51^ In the nucleus, histone-modifying enzymes, such as SETDB1, SUV39H2, and KMT2A were also associated with areca nuts, suggesting that areca nut-induced mutations also deregulate the expression and silencing of various genes that can promote oral cancer development and progression.^54^^,^^55^

IL-6 as a hub protein in the areca nut-associated PPI network is related to inflammatory lymphangiogenesis in OSCC. IL-6 production is induced by areca nuts, which leads to the development of oral OSF by stimulating the proliferation of oral epithelial cells and mucosal inflammation.^56^^,^^57^ Upon activation, IL-6 also stimulates the JAK/STAT, PI3K, and MAPK pathways, which mediate inflammation and cancer development. In response to areca nut exposure, IL-6 induces the production of necrotic and inflammatory cytokines, which aid in the progression of oral cancer and possibly its malignant transformation.^58^ Cytokine activity was associated with both areca nut- and nicotine-related proteins [Supplementary Figure 7].

Analyzing network clusters could be another approach for the development of therapeutics [Supplementary Tables 2 and 3]. The largest cluster of areca nut-associated proteins contained all five hub proteins. Physiological analysis of the largest clusters showed that the same organs, systems, and regions of the body were affected by areca nut and nicotine use [Supplementary Table 4]. An association has been found between areca nut consumption and head and neck cancer. The expression sites associated with areca nut related to the oral cavity were the oral mucosa, esophagus, salivary glands, saliva, and tonsils [Supplementary Figure 8]. In addition, areca nuts affect the parathyroid glands, whereas nicotine affects the adrenal glands. Intriguingly, the reproductive system is among the most affected by areca nuts; however, limited research is available on this aspect [Supplementary Table 4].

Although most biological processes affected by areca nuts and nicotine were common, some pathways, such as extracellular matrix organization and lipid storage and metabolism, were only affected by areca nut exposure, which can be used to discover differential markers of areca nut exposure [Supplementary Figures 4–6]. Similarly, CDK activity was present in areca nut-associated proteins but absent in nicotine-associated proteins. Areca nut-associated proteins were also uniquely localized in chromosomes, neuron projections, and synapses, but not so much in the cranial nerves [Figure 2].

One of the limitations of this study is that no weight was assigned to any protein based on the expression level. Thus, all proteins were considered to be associated with areca nut exposure, and interpretations were made based on their role in cancer. The second limitation is that the study compared proteins associated with areca nuts, rather than specific alkaloids. Owing to the limited knowledge on areca nut alkaloids, studying the effect of a single alkaloid on the human body may not present accurate unbiased results.

## Conclusions

Network analysis of systematically collated proteins associated with areca nut use suggested that areca nuts induce toxicity by affecting regulatory mechanisms that systemically affect multiple body parts. Ontology-based characterization of proteins indicated that hub proteins are pivotal in regulating the cell signaling mechanisms leading to toxicity and oncogenesis. Although the molecular effects of the areca nut largely overlap with those of nicotine, suggesting a similar mechanism of toxicity induction, the association of areca nut with some unique pathways could be the basis for the identification of potential markers of areca nut exposure and targets for anticancer therapeutic agents. Thus, extensive evaluation of the unique proteins and associated pathways need to be evaluated.

## Funding

This work was supported by intramural funding from the National Institute of Cancer Prevention and Research, Indian Council of Medical Research (No. NICPR/Anuj/intramural/2021/1).

## Authors contribution

Anuj Kumar: Conceptualization, Investigation, Writing – original draft, Supervision, Writing – review & editing; Krati Garg: Investigation, Writing – original draft; Vidisha K: Investigation. Pramod Kumar: Writing – review & editing; Shalini Singh: Writing – review & editing.

## Ethics statement

None.

## Data availability statement

The data and materials for this study are available from the corresponding author upon reasonable request.

## Conflict of interest

The authors declare that they have no known competing financial interests or personal relationships that could have appeared to influence the work reported in this paper.
