# Supplementary material for: Overlap in oncogenic and pro-inflammatory pathways associated with areca nut and nicotine exposure
Source: Cancer Pathog Ther. 2023 Sep 17;2(3):187–94. doi: 10.1016/j.cpt.2023.09.003 (PMC11252521; doi:10.1016/j.cpt.2023.09.003)
Supplement: Multimedia component 1 [file mmc1.docx]

***Appendix A. Supplementary data***

**Figure Legends**

**
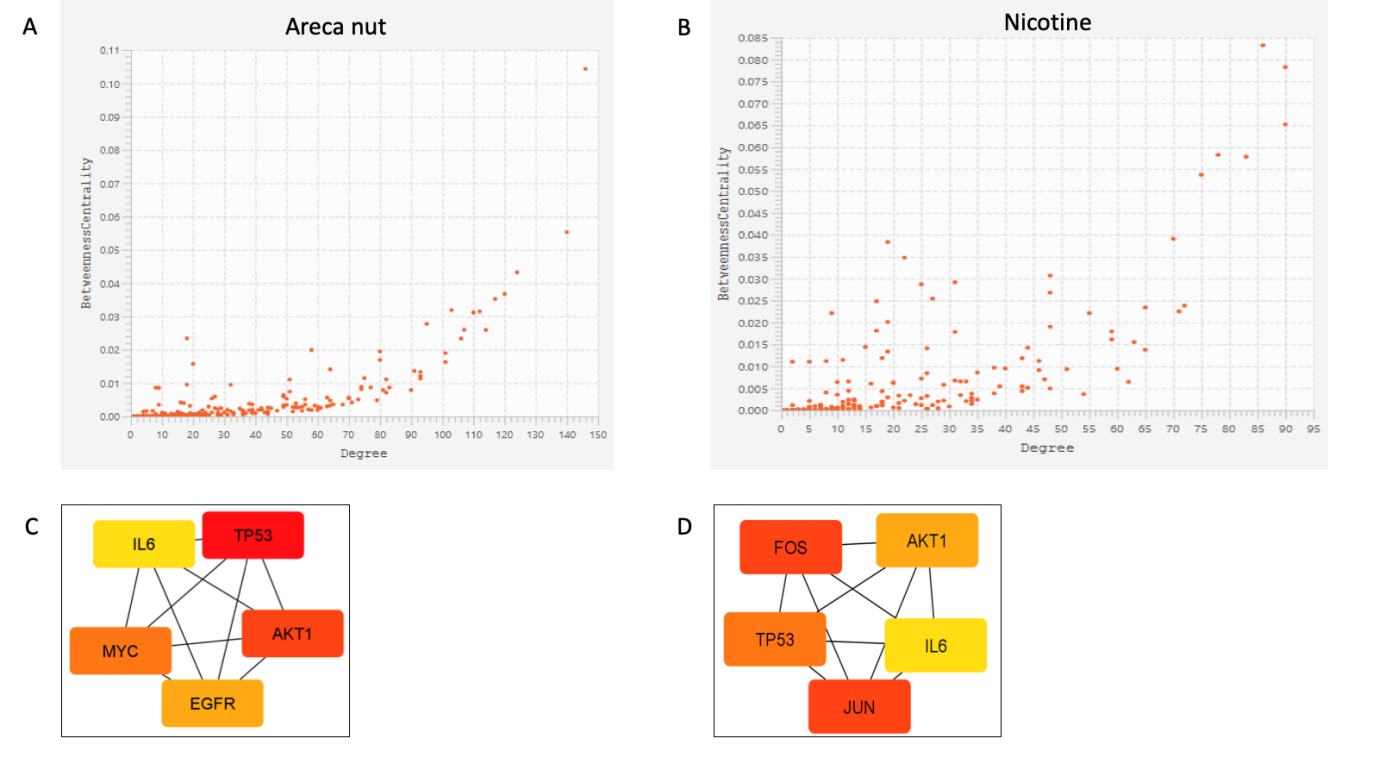
**

**Supplementary Figure 1:** Betweenness by degree of the PPI networks. (A and B) (A) Areca nut and (B) nicotine-associated proteins determined using the Cytoscape tool. (C) Major hub proteins of the PPI network were determined using the CyChart tool of Cytoscape. (D) Hub proteins of areca nut PPI network. Hub proteins of nicotine PPI network. Major hub proteins are connected to each other. AKT1: Alpha serine/threonine-protein kinase; EGFR: Epidermal growth factor receptor; IL6: Interleukin-6; JUN; MYC: Master regulator of cell cycle entry and proliferative metabolism; PPI: Protein-protein interaction; TP53: Tumor protein 53.


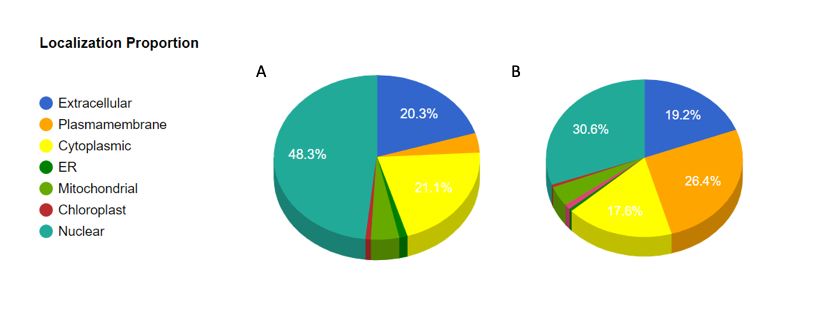


**Supplementary Figure 2:** Subcellular protein localization. (A) Areca nut-associated proteins (226 proteins) (B) Nicotine-associated proteins (200 proteins) determined using the subCELlular LOcalization prediction with annotation of functional Gene Ontology prediction tool. ER: Endoplasmic reticulum.


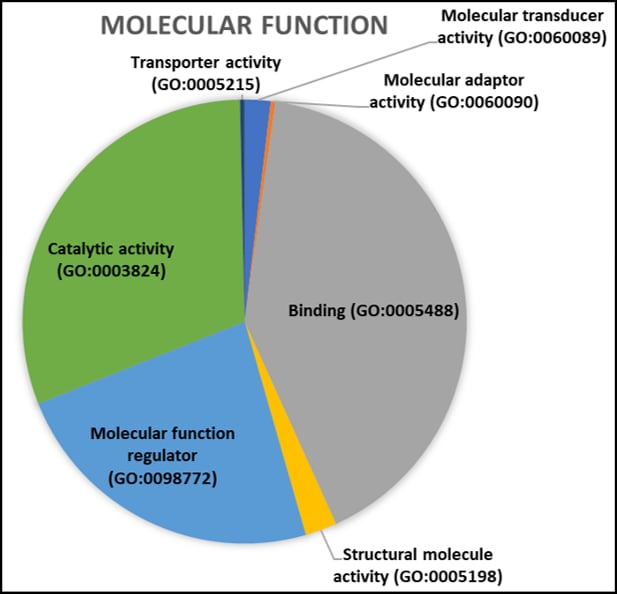

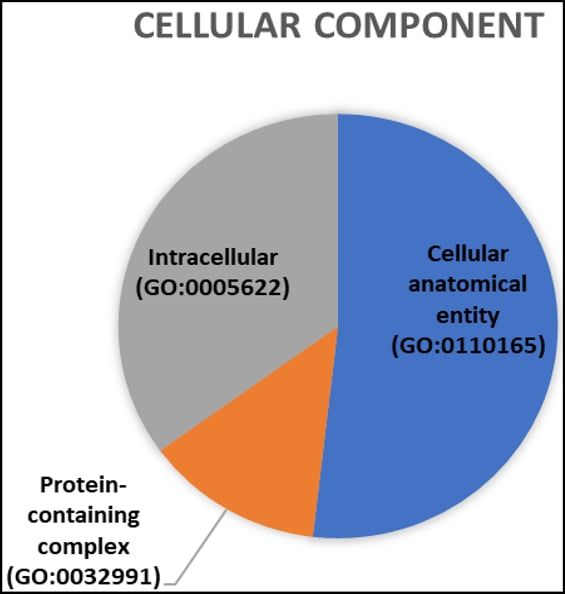

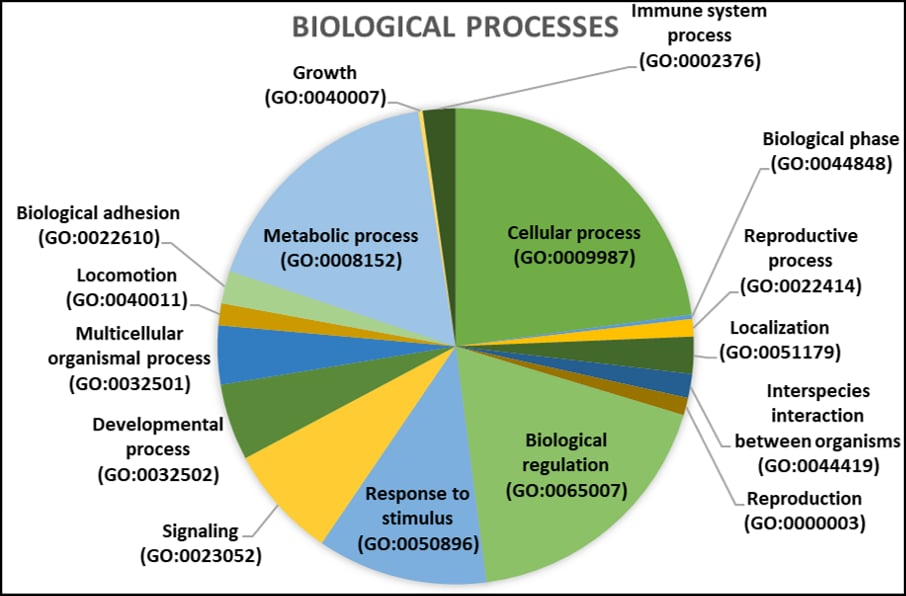

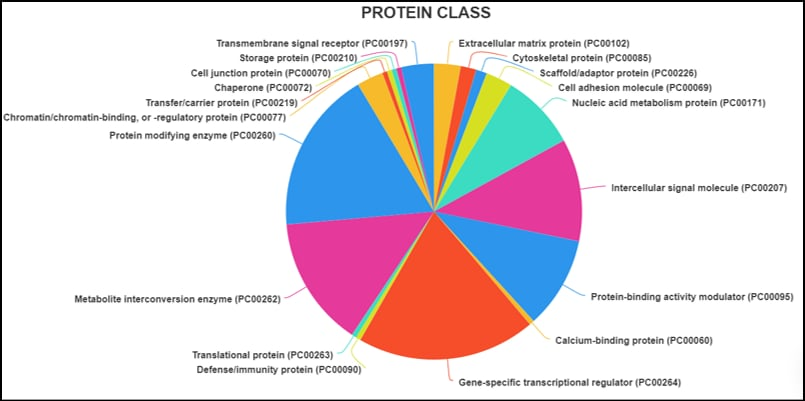

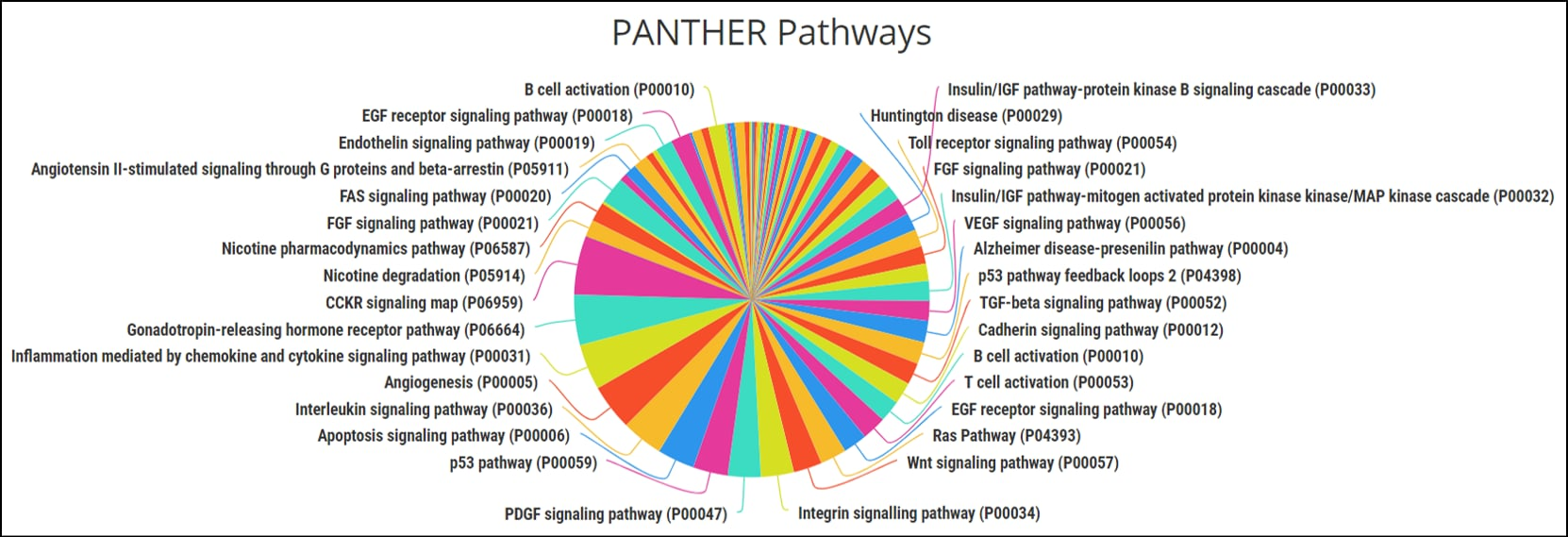


A

B

D

C

E

**Supplementary Figure 3:** Functional analysis of PPI network of proteins associated with areca nut using the protein analysis through evolutionary relationships (PANTHER) classification system. B cell: Bursa-derived cells; CCKR: Cholecystokinin receptor; EGF: Epidermal growth factor; FAS: FS-7-associated surface antigen; FGF: Fibroblast growth factors; IGF: Insulin-like growth factor; PANTHER: Protein analysis through evolutionary relationships; PDGF: Platelet-derived growth factor; PPI: Protein-protein interaction; Ras: Rat sarcoma; T cell: Thymus cells; TGF: Transforming growth factor; VEGF: Vascular endothelial growth factor; Wnt: Wingless/integrated.


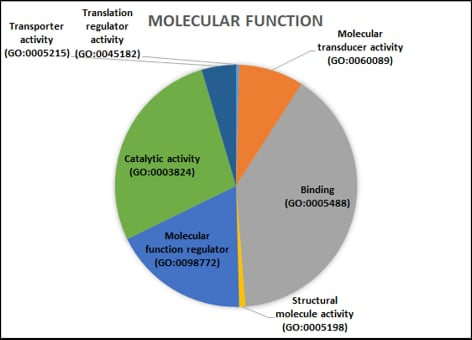

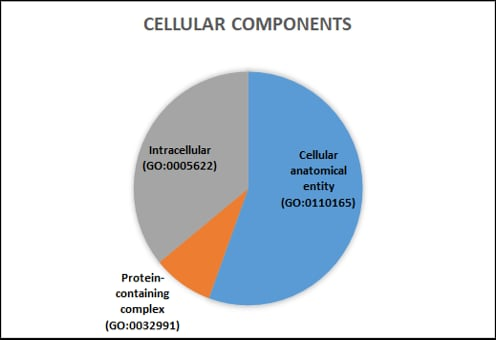

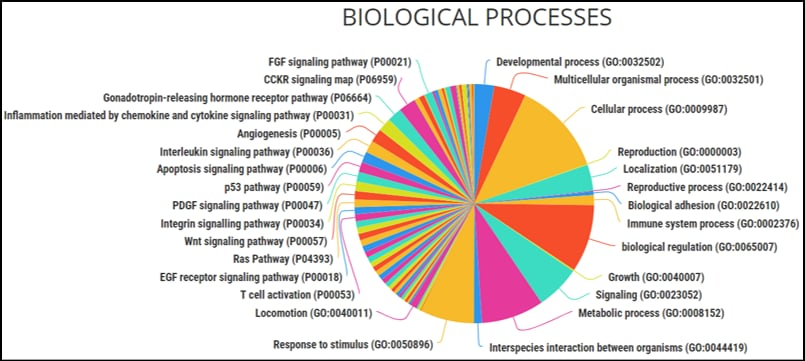

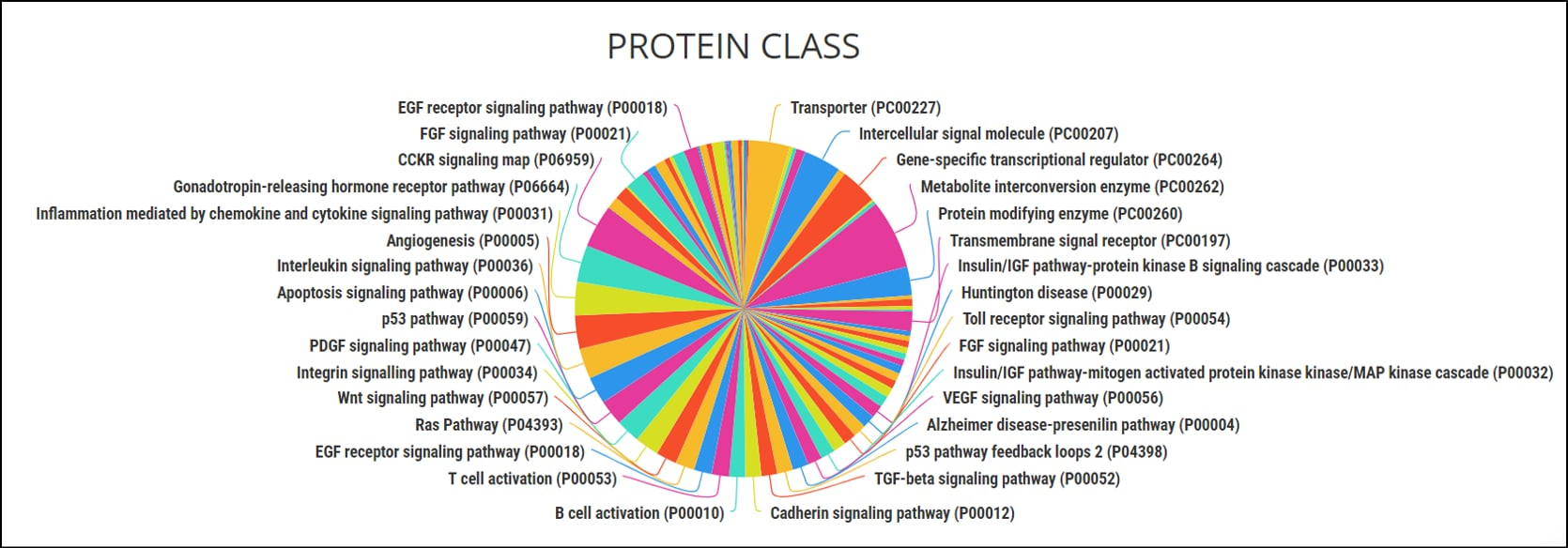

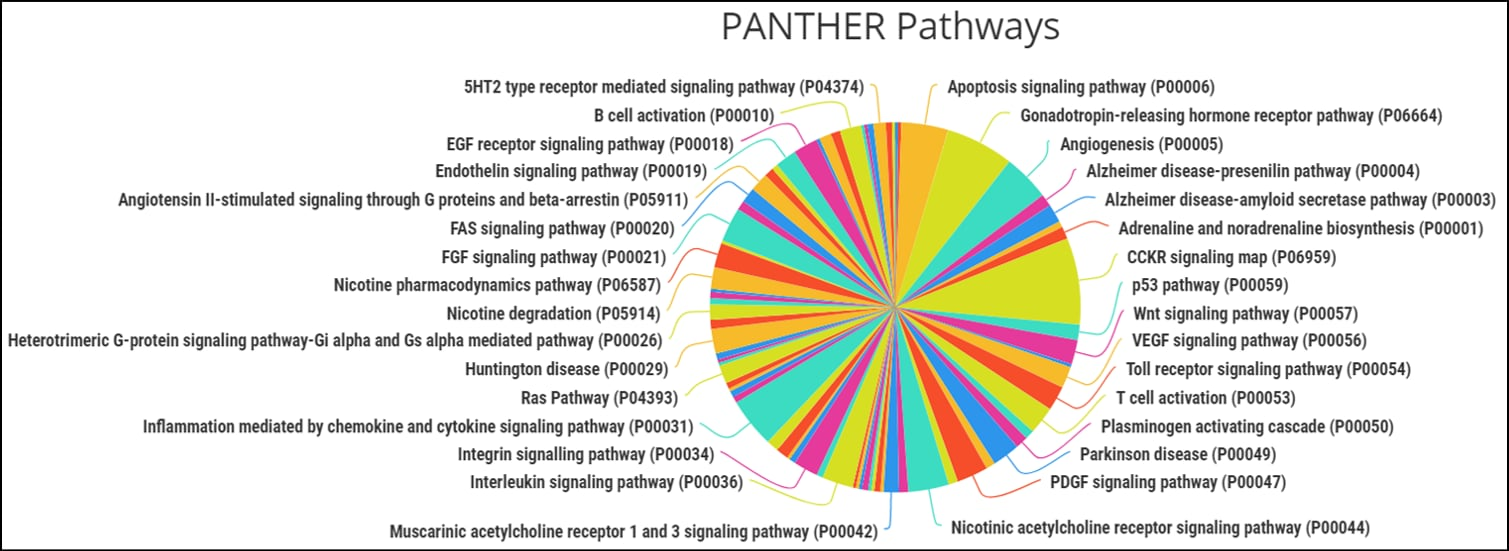


A

B

D

C

E

**Supplementary Figure 4:** Functional analysis of PPI network of proteins associated with nicotine using the Protein ANalysis THrough Evolutionary Relationships (PANTHER) classification system. B cell: Bursa-derived cells; CCKR: Cholecystokinin receptor; EGF: Epidermal growth factor; FAS: FS-7-associated surface antigen; FGF: Fibroblast growth factors; IGF: Insulin-like growth factor; PANTHER: Protein analysis through evolutionary relationships; PDGF: Platelet-derived growth factor; PPI: Protein-protein interaction; Ras: Rat sarcoma; T cell: Thymus cell; TGF: Transforming growth factor; VEGF: Vascular endothelial growth factor; Wnt: Wingless/integrated.

**
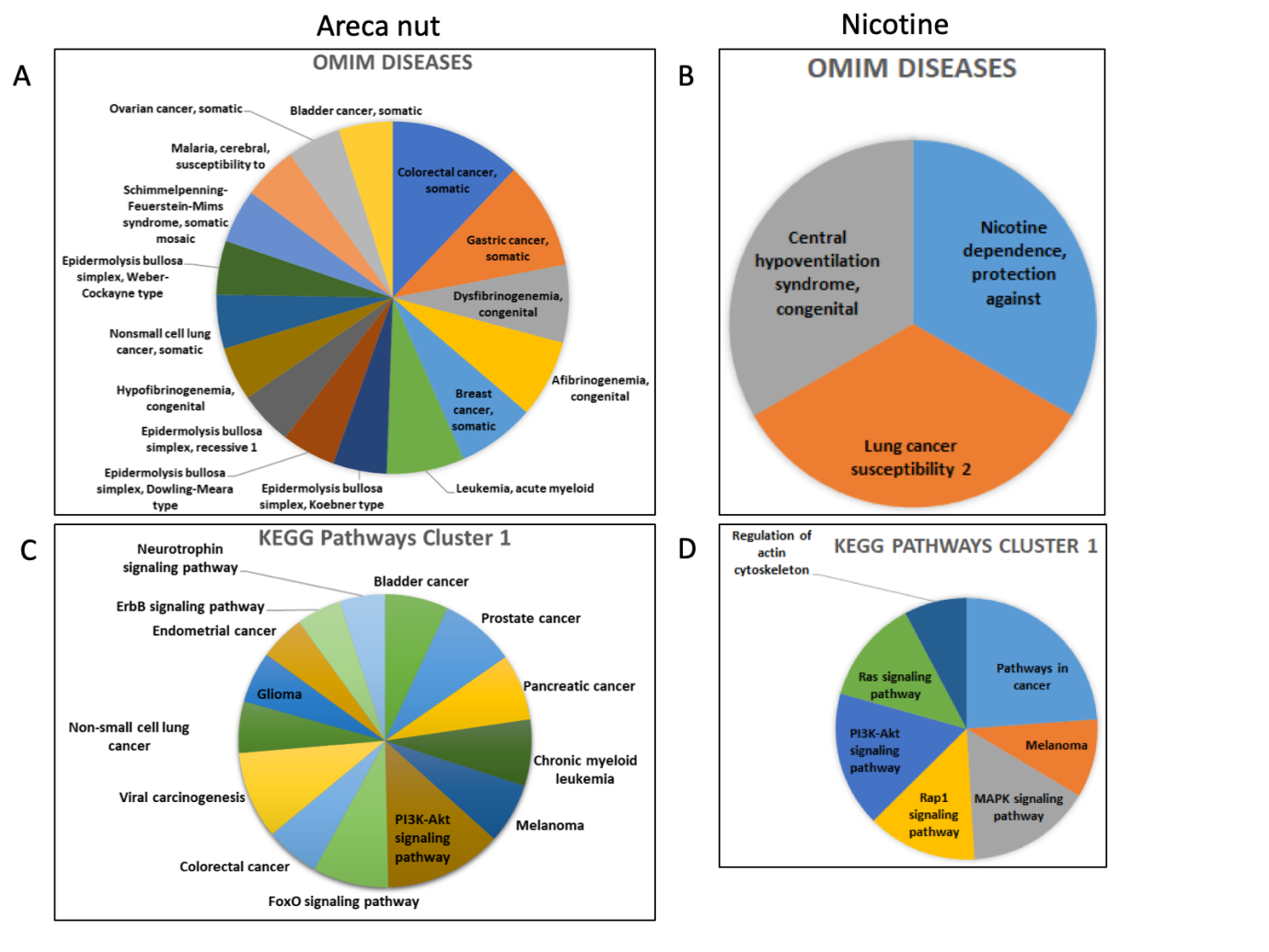
**

**Supplementary Figure 5:** Role of proteins in metabolism and human disease. (A and B) (A) Online Mendelian Inheritance in Man (OMIM) and (B) Kyoto Encyclopedia of Genes and Genomes (KEGG) pathway (cluster 1) classification of areca nut-associated proteins. (C and D) (C) Online Mendelian Inheritance in Man (OMIM) disease and (D) Kyoto Encyclopedia of Genes and Genomes (KEGG) pathway (cluster 1) classification of nicotine-associated proteins. FoxO: Forkhead box O; KEGG: Kyoto Encyclopedia of Genes and Genomes; MAPK: Mitogen-activated protein kinase; OMIM: Online Mendelian Inheritance in Man; PI3K: Phosphoinositide-3-kinase; Rap1: Repressor/activator protein 1; Ras: Rat sarcoma.

*
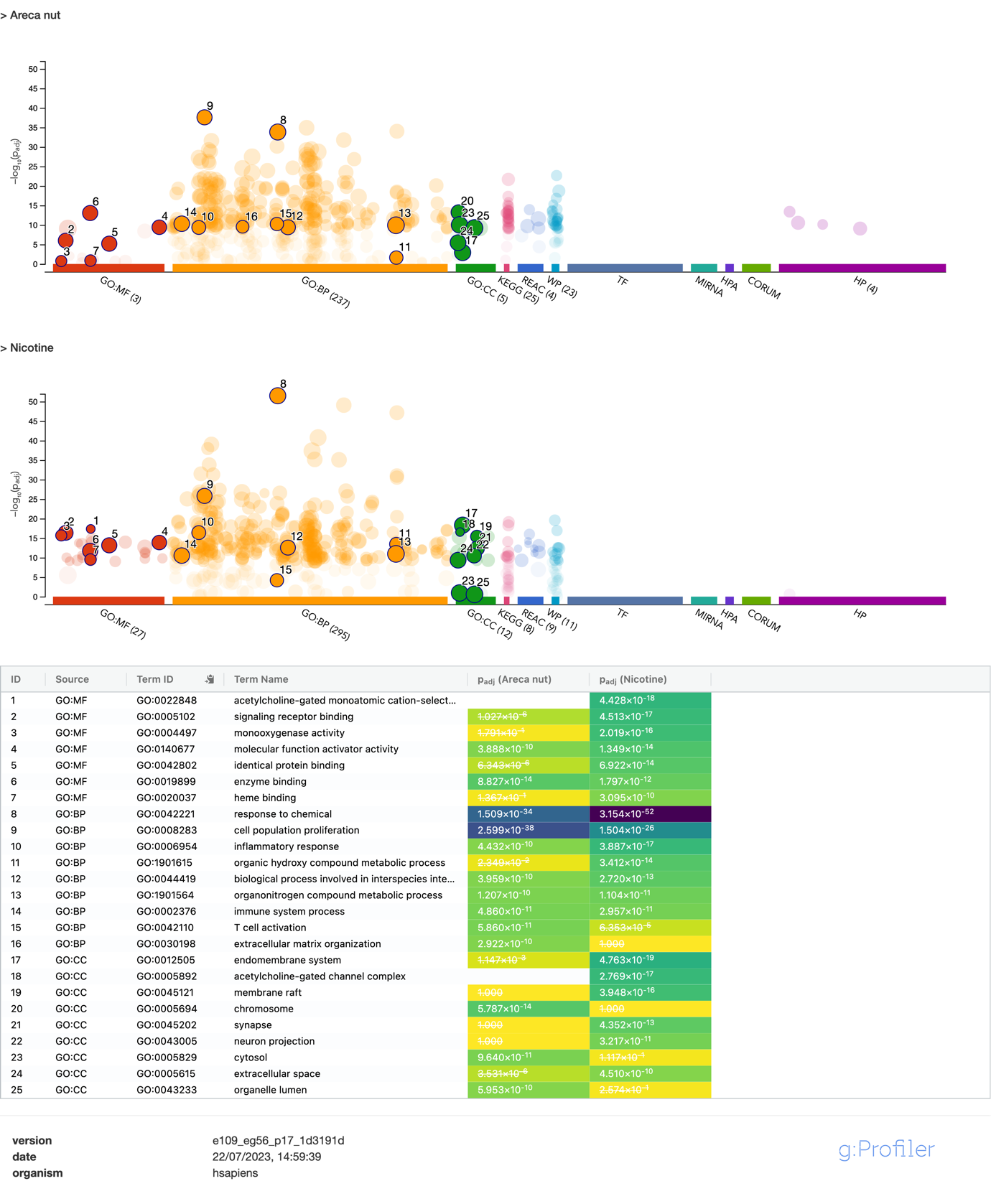
*

**Supplementary Figure 6:** Comparative g:profiler, g:HOST analysis of areca nut-associated proteins and nicotine-associated proteins at *P* < 10^−9^. Three subsections of Gene Ontology (GO) are shown, namely molecular function (MF) performed by the protein, biological process (BP) in which the protein is involved, and cellular component (CC) where the protein is localized. In addition, data from Kyoto Encyclopedia of Genes and Genomes (KEGG), Reactome (REAC), WikiPathways (WP), TRANSFAC (TF), mirTarBase (MIRNA), Human Protein Atlas (HPA), CORUM, and Human Phenotype Ontology (HPO) are shown. BP: Biological process; CC: Cellular component; CORUM: Comprehensive resource of mammalian protein complexes; GO: Gene Ontology; HP: Human Phenotype Ontology; HPA: Human Protein Atlas; KEGG: Kyoto Encyclopedia of Genes and Genomes; MF: Molecular function; MIRNA: mirTarBase; REAC: Reactome; TF: TRANSFAC; WP: WikiPathways.

*
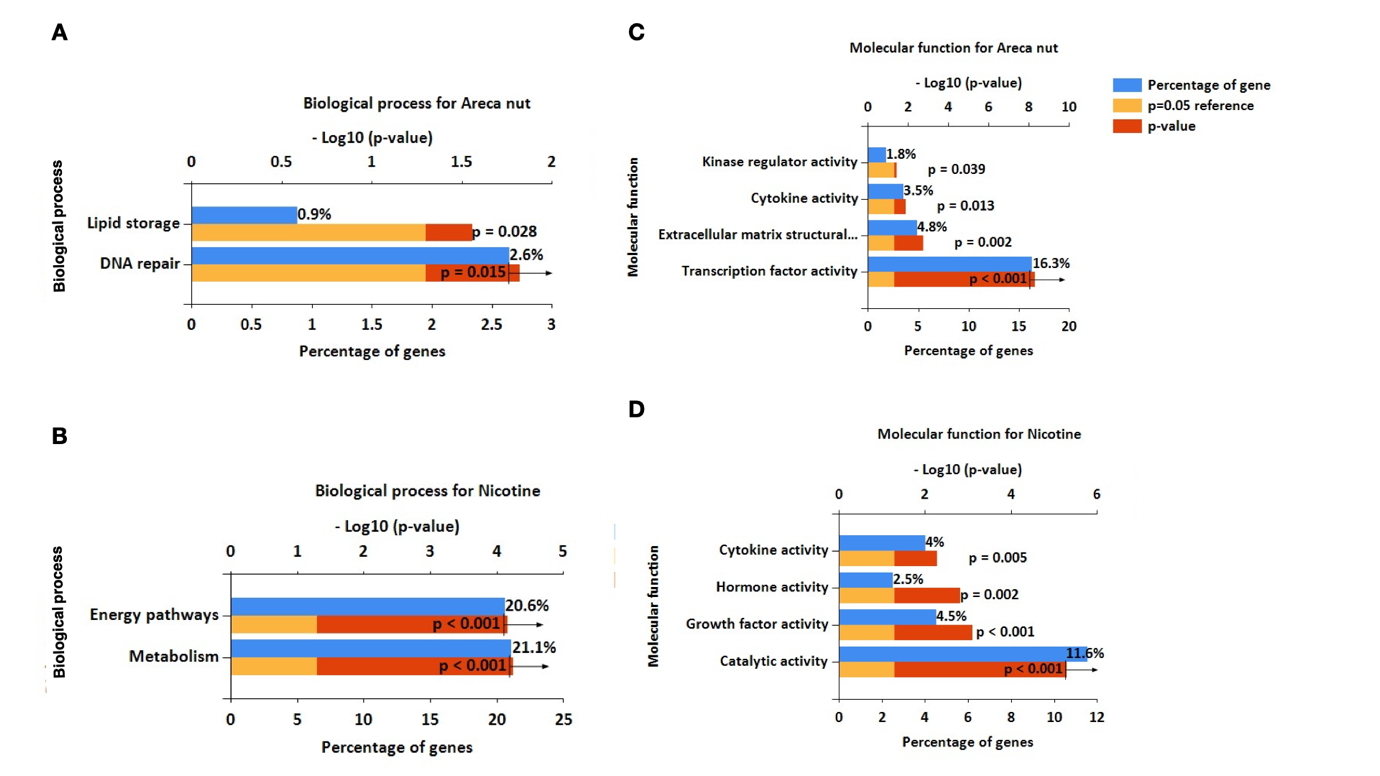
*

**Supplementary Figure 7:** Enrichment analysis of areca nut- and nicotine-associated proteins at *P* < 0.05. Biological processes associated with (A) areca nut and (B) nicotine. Molecular functions associated with (C) areca nut and (D) nicotine. DNA: Deoxyribonucleic acid.

*
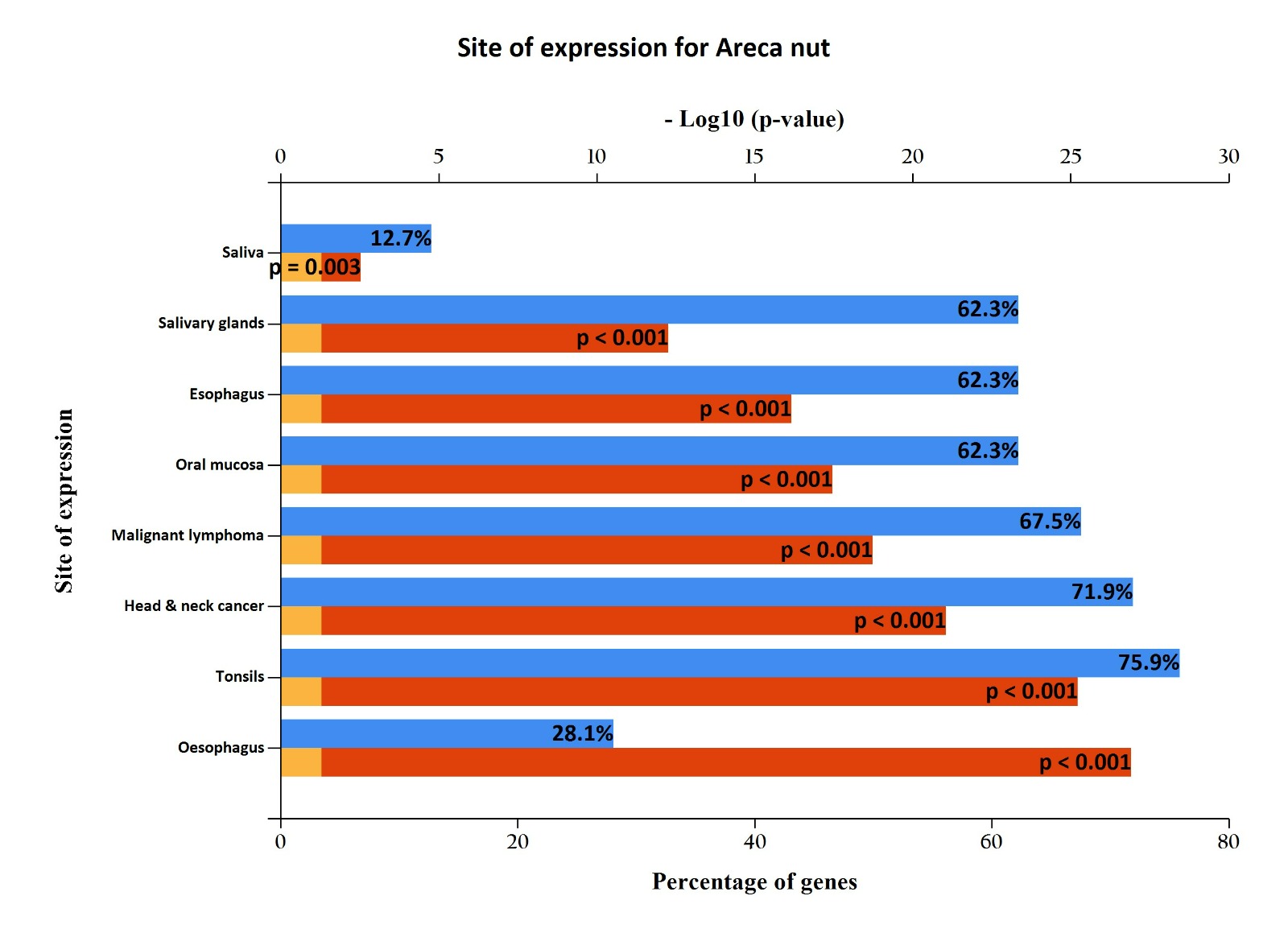
*

**Supplementary Figure 8:** Enrichment analysis of expression site for areca nut-associated proteins using FunRich.

*
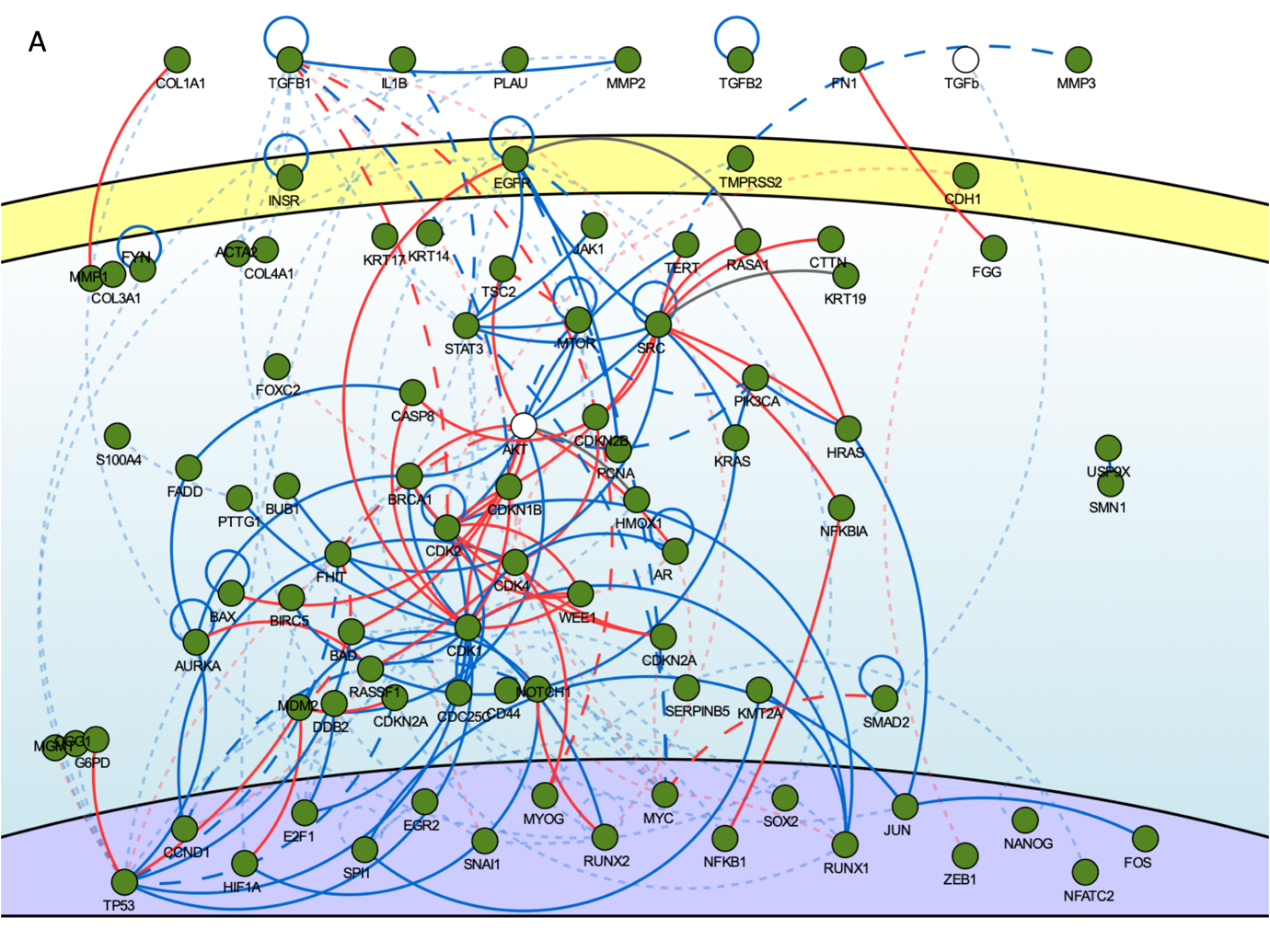

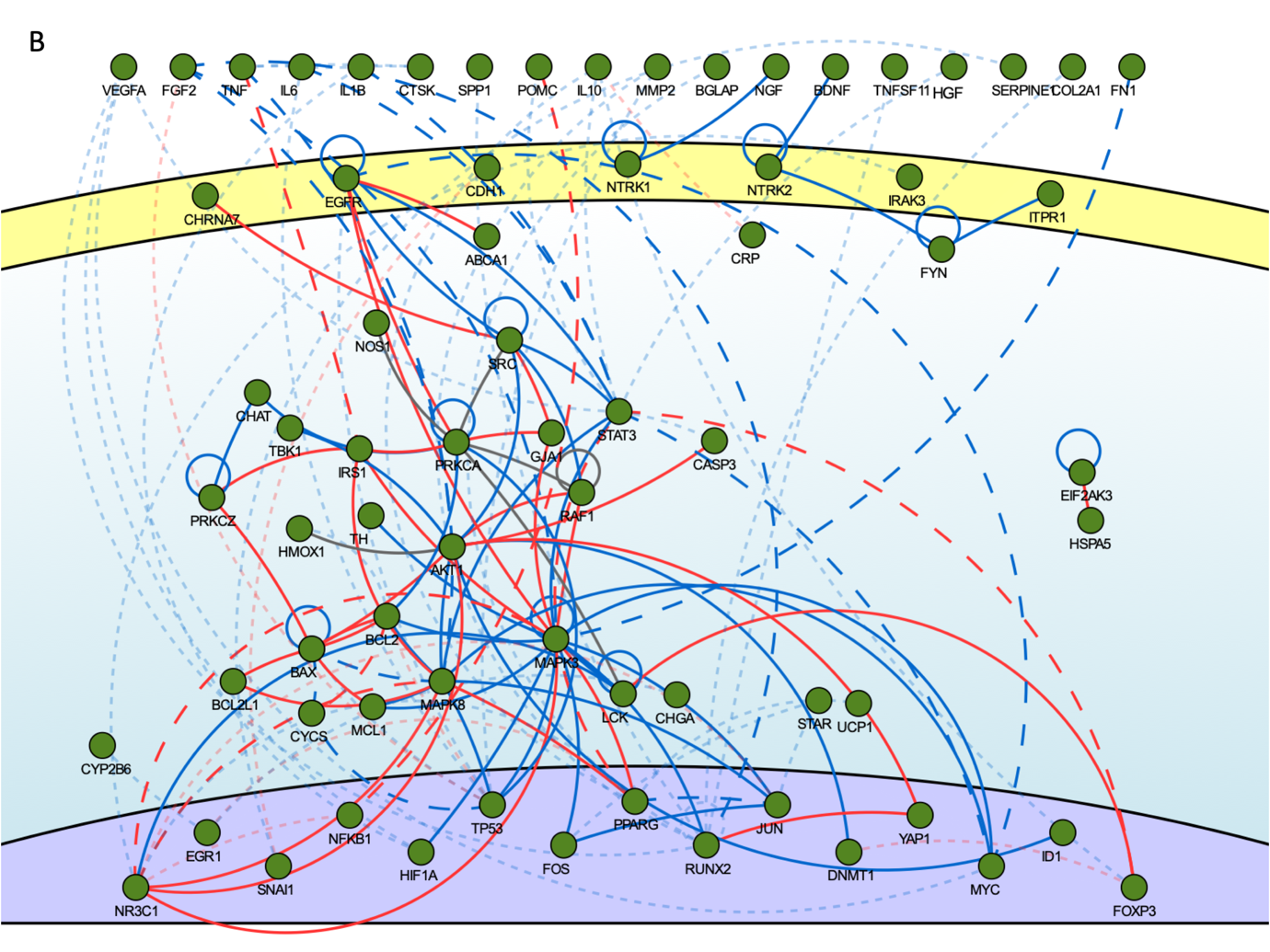
***Supplementary Figure 9:** Analysis of signaling interactions between proteins and their biological relevance’s. Signaling interaction between (A) areca nut-associated proteins and (B) nicotine-associated proteins. In the diagram, proteins and small molecules are spatially organized in four main cellular compartments: extracellular space, plasma membrane, cytoplasm, and nucleus, according to their manual annotation. Color code: small dark-green circles represent nodes (proteins) used to query the SIGNOR database and retrieve the interactions that form the graph; light green represents first neighbors. White circles and blue clover leaves represent protein families and complexes, respectively. Direct interactions are displayed as solid lines, and indirect ones are dashed lines. Edge color and arrow shape represent the effect (up- or downregulation): blue arrows represent upregulation, and red “T-shaped” arrows represent downregulation. SIGNOR: Signaling network open resource

*
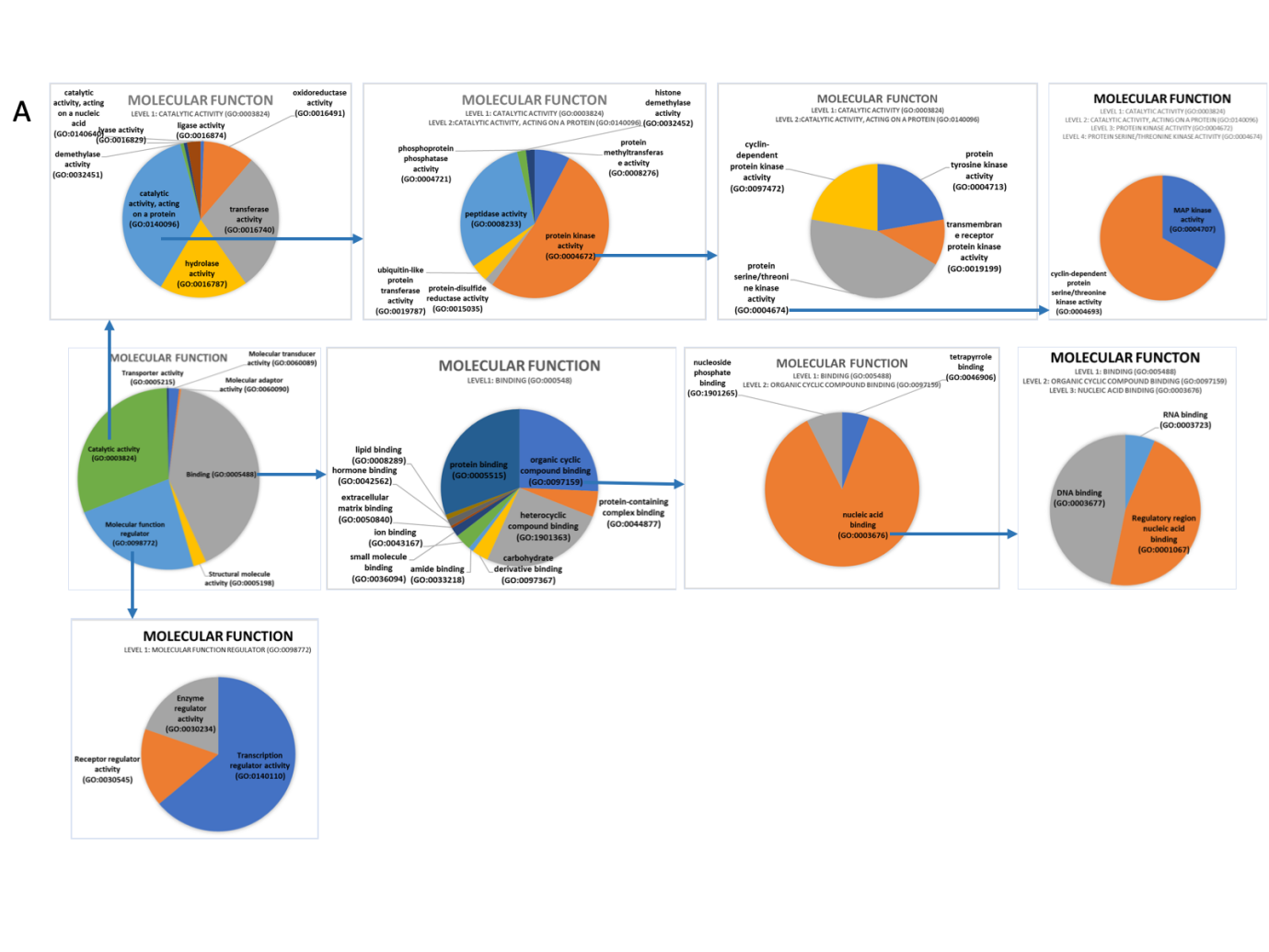
*

*
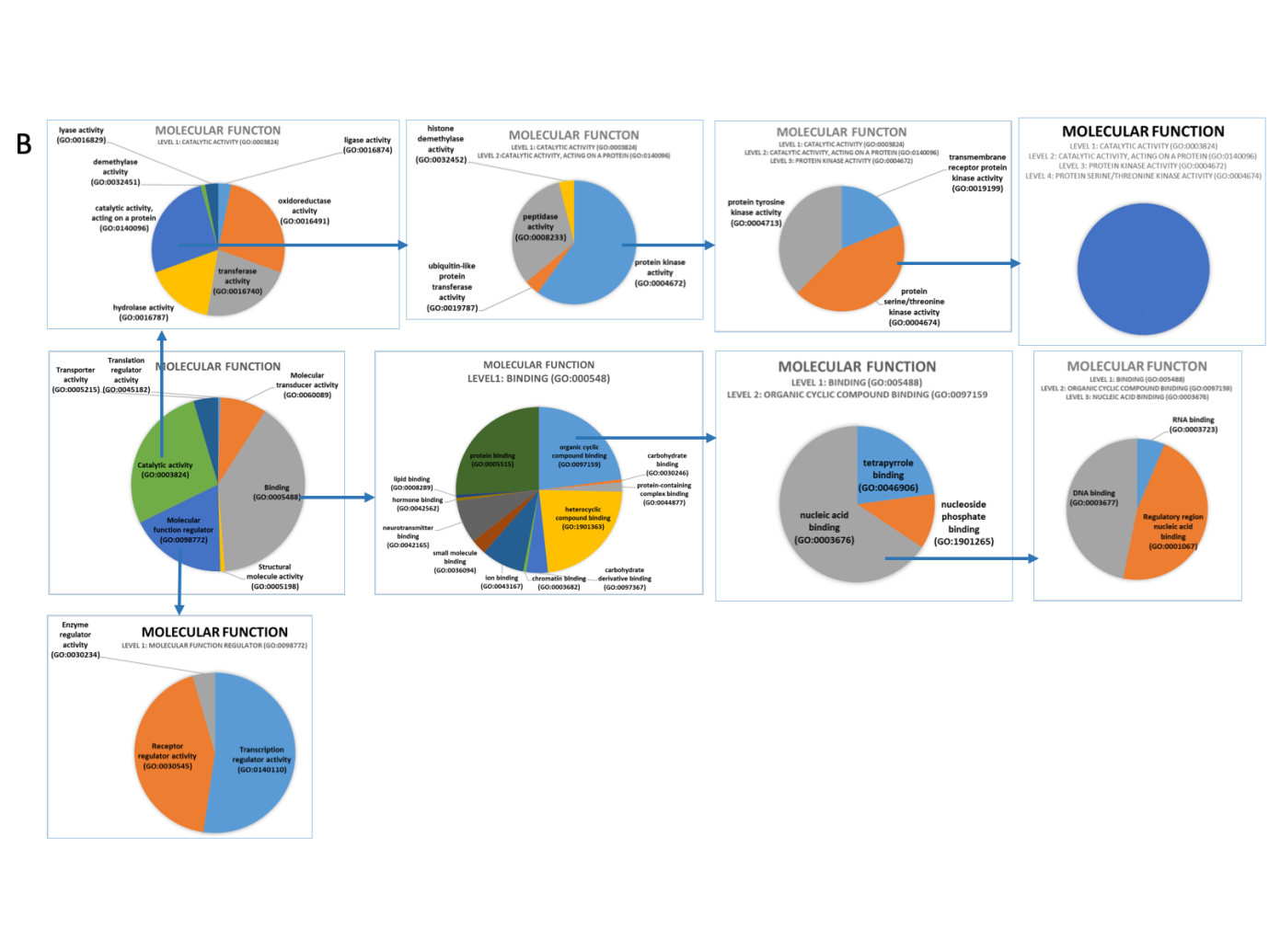
*

**Supplementary Figure 10:** In-depth molecular function analysis of proteins. (A) areca nut-associated proteins and (B) nicotine-associated proteins using pie-of-pie charts. A pie chart of major pies was prepared for in-depth analysis.

**Supplementary Table 1**: **Topological parameters of the networks created for areca nut and nicotine-associated proteins.**

| **Attributes** | **Areca nut network** | **Nicotine network** |
| --- | --- | --- |
| Number of proteins | 225 | 201 |
| Number of edges | 3994 | 2206 |
| Average number of neighbors | 36,475 | 21,950 |
| Network diameter | 5 | 2 |
| Network radius | 3 | 1 |
| Characteristic path length | 2.040 | 1.890 |
| Clustering coefficient | 0.598 | 0.619 |
| Network density | 0.167 | 0.110 |
| Network heterogenicity | 0.845 | 1.098 |
| Network centralization | 0.507 | 0.899 |
| Connected components | 7 | 1 |

**Supplementary Table 2:** **MCODE clusters of the areca nut-associated PPI network.**

| **Cluster** | **Nodes** | **Edges** | **MCODE score** | **Genes** |
| --- | --- | --- | --- | --- |
| C1 | 63 | 1470 | 47,419 | *ZEB1, VCAM1, TP53, TNF, TIMP2, TIMP1, TGFB1, TERT, STAT3, STAT1, SRC, SOX9, SOX2, SNAI2, SNAI1, SMAD2, SERPINE1, RUNX2, PTGS2, POU5F1, PLAU, PI3, NOTCH1, NANOG, MYC, MTOR, MMP9, MMP3, MMP2, MMP1, MDM2, MAPK3, MAPK1, LOX, KRAS, JUN, IL-6, IL1B, IL10, IGF1R, IGF1, ICAM1, HRAS, HMOX1, HIF1A, FYN, FOS, FN1, ERBB2, EGFR, CXCL8, CTGF, CDKN2A, CDKN1B, CDKN1A, CDH1, CD44, CCND1, CCNB1, CASP8, BRCA1, AR, AKT1* |
| C2 | 7 | 17 | 5667 | *TTK, PTTG1, PCNA, MAD2L1, BUB1B, BUB1, APC* |
| C3 | 7 | 17 | 5667 | *SULT1A1, NAT2, G6PD, CYP3A5, CYP2E1, CYP2A6, CYP1A2* |
| C4 | 17 | 40 | 5000 | *WNT5A, TSC2, TNFSF11, RELA, PIK3CA, NFKBIA, NFKB1, JAK1, IRS1, IL18, HSPB1, FGF7, ETS1, CDK4, CDK2, CDK1, BRCA2* |
| C5 | 23 | 47 | 4273 | *WEE1, TGFB2, SPARC, RASSF1, OSM, MGMT, KRT5, KRT19, KRT14, IRF1, IL1A, H2AFX, FHIT, E2F1, CYR61, COL1A1, CDKN2B, CDC25C, CCR2, CCL4, CASP1, BIRC5, AURKA* |
| C6 | 5 | 8 | 4000 | *SND1, HSP90B1, FGG, FGB, FGA* |
| C7 | 4 | 6 | 4000 | *XRCC4, XRCC3, XRCC1, OGG1* |
| C8 | 4 | 5 | 3333 | *ITGAV, COL4A1, COL3A1, ACTA2* |

MCODE: Molecular complex detection; PPI: Protein-protein interaction.

**Supplementary Table 3: MCODE clusters of nicotine-related PPI network.**

| **Cluster** | **Nodes** | **Edges** | **MCODE score** | **Genes** |
| --- | --- | --- | --- | --- |
| C1 | 28 | 273 | 20,222 | *AKT1, BAX, BCL2, CASP3, EIF2AK3, FGF1, FGF13, FOS, GJA1, HSPA4, HSPA5, IRS1, JUN, MCL1, MYC, MYD88, NFKB1, NGF, NR3C1, PDGFB, POMC, RAF1, SRC, STAT3, TNF, TP53, VEGFA* |
| C2 | 29 | 266 | 19,000 | *AGT, BCL2L1, BDNF, CDH1, CSF2, EGFR, EGR1, FGF2, FOXP3, FYN, HIF1A, HMOX1, IL10, IL1B, IL2, IL6, IL8, LCK, MAPK3, MAPK8, MMP2, MMP9, NOS1, PPARG, PTGS2, RUNX2, SERPINE1, SNAI1, TF* |
| C3 | 10 | 45 | 10,000 | *CHRNA1, CHRNA7, CHRNA6, CHRNA4, CHRNB3, CHRNB2, CHRNA3, CHRNA2, CHRNB4, CHRNA5* |
| C4 | 18 | 68 | 8000 | *SLC6A3, HTR7, CYP1A2, UGT1A4, UGT1A9, CYP3A4, CYP1A1, CRH, CYP2E1, HCRT, UGT1A3, HTR2A, CYP2A13, AVP, HTR3A* |
| C5 | 6 | 10 | 4000 | *NTRK2, NPY, PNMT, CHAT, CCK, CNR1* |
| C6 | 3 | 3 | 3000 | *PON1, ABCA1, LDLR* |

MCODE: Molecular complex detection; PPI: Protein-protein interaction.

**Supplementary Table 4: Phenotypic analysis of the largest cluster (cluster 1) of the areca nut and nicotine PPI networks.**

| **Protein enrichment** | **Areca nut-associated proteins** | **Nicotine-associated proteins** |
| --- | --- | --- |
| **Organs** | *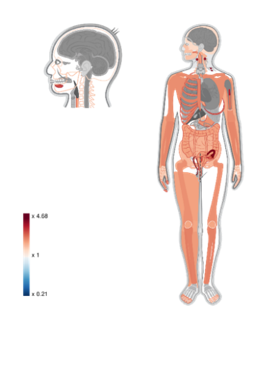*  67 of 125 organs | 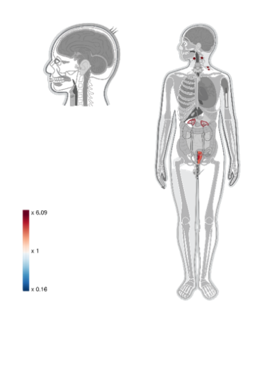  21 of 125 organs |
| Vas deferens | 4.6781, *P* = 0.0002 | NA |
| Parathyroid | x4.3771, *P* = 0.064 | NA |
| Adrenal glands | NA | x4.49, *P* = 0.0004 |
| Fallopian tube | x4.1288, *P* = 0.0027 | NA |
| Uterus | NA | x2.66, *P* = 0.0011 |
| Lymph nodes | x3.1716, *P* = 0.0026 | x6.09, *P* = 0.0003 |
| **Systems** | *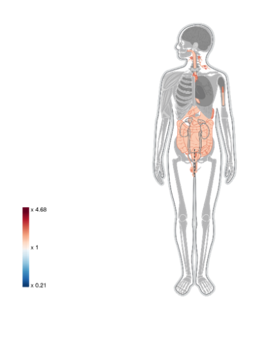*  3/6 body regions | 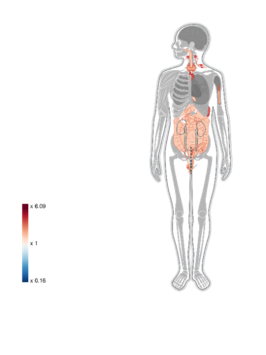  2/6 body regions |
| Lymphatic system | x1.8368, *P* = 0.00426 | x2.77, *P* = 0.0008 |
| Endocrine system | x1.4865, *P* = 0.0283 | x1.93, *P* = 0.0184 |
| Immune system | x1.4502, *P* = 0.00428 | x1.85, *P* = 0.0015 |
| Reproductive system | NA | x1.72, *P* = 0.0179 |
| Digestive system | NA | x1.50, *P* = 0.0159 |
| **Region** | *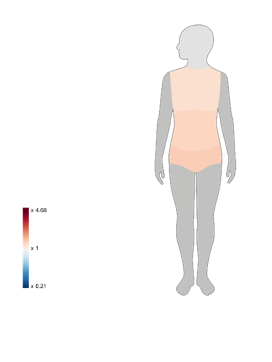*  7/12 body systems | 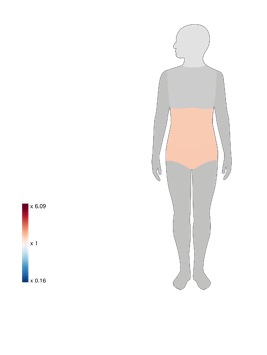  5/12 body systems |
| Pelvis | x1.3991, *P* = 0.00168 | x1.5250, *P* = 0.0131 |
| Abdomen | x1.3255, *P* = 0.00387 | x1.4970, *P* = 0.00916 |
| Thorax | x1.2131, *P* = 0.0197 | NA |

The heat map shows the level of enrichment; the darker red color indicates higher enrichment. x stands for fold increase in comparison to the normal level of the protein. PPI: Protein-protein interaction.

**Supplementary Table 5: The most enriched genes in cluster 1 of the areca nut protein-protein interaction (PPI) network in different body parts.**

| **Gene name** | **Body parts affected** |
| --- | --- |
| *KRAS* | Lymph nodes, fallopian tube, vas deferens, abdomen, thorax, pelvis, immune system, lymphatic system, and endocrine system |
| *CDKN1A* | Lymph nodes, parathyroid, abdomen, thorax, pelvis, immune system, lymphatic system, and endocrine system |
| *CDKN1B* | Lymph nodes, parathyroid, abdomen, thorax, pelvis, immune system, lymphatic system, and endocrine system |
| *AKT1* | Lymph nodes, parathyroid, abdomen, thorax, pelvis, immune system, lymphatic system, and endocrine system |
| *TGFB1* | Fallopian tube, vas deferens, abdomen, thorax, pelvis, immune system, lymphatic system, and endocrine system |
| *STAT1* | Fallopian tube, vas deferens, abdomen, thorax, pelvis, immune system, lymphatic system, and endocrine system |
| *STAT3* | Fallopian tube, vas deferens, abdomen, thorax, pelvis, immune system, lymphatic system, and endocrine system |

PPI: Protein-protein interaction.

**Supplementary Table 6: The most enriched genes in cluster 1 of the nicotine protein-protein interaction (PPI) network in various body parts.**

| **Gene name** | **Body parts affected** |
| --- | --- |
| *AKT1* | Lymph nodes, adrenal glands, uterus, abdomen, pelvis, immune system, lymphatic system, endocrine system, reproductive system, and digestive system |
| *POMC* | Lymph nodes, adrenal glands, uterus, abdomen, pelvis, immune system, lymphatic system, endocrine system, reproductive system, and digestive system |
| *NFKB1* | Lymph nodes, adrenal glands, uterus, abdomen, pelvis, immune system, lymphatic system, endocrine system, reproductive system, and digestive system |
| *RAF1* | Lymph nodes, adrenal glands, uterus, abdomen, pelvis, immune system, lymphatic system, endocrine system, reproductive system, and digestive system |
| *TP53* | Adrenal glands, uterus, abdomen, pelvis, immune system, lymphatic system, endocrine system, reproductive system, and digestive system |
| *EIF2AK3* | Adrenal glands, uterus, abdomen, pelvis, immune system, lymphatic system, endocrine system, reproductive system, and digestive system |

PPI: Protein-protein interaction.
